# Facultative heterochromatin formation in rDNA is essential for cell survival during nutritional starvation

**DOI:** 10.1093/nar/gkac175

**Published:** 2022-03-28

**Authors:** Hayato Hirai, Naomichi Takemata, Miki Tamura, Kunihiro Ohta

**Affiliations:** Department of Life Sciences, Graduate School of Arts and Sciences, The University of Tokyo, Komaba 3-8-1, Tokyo 153-8902, Japan; Department of Life Sciences, Graduate School of Arts and Sciences, The University of Tokyo, Komaba 3-8-1, Tokyo 153-8902, Japan; Department of Life Sciences, Graduate School of Arts and Sciences, The University of Tokyo, Komaba 3-8-1, Tokyo 153-8902, Japan; Department of Life Sciences, Graduate School of Arts and Sciences, The University of Tokyo, Komaba 3-8-1, Tokyo 153-8902, Japan; Universal Biology Institute, The University of Tokyo, Hongo 7-3-1, Tokyo 113-0033, Japan

## Abstract

During the cellular adaptation to nutrient starvation, cells temporarily decelerate translation processes including ribosomal biogenesis. However, the mechanisms repressing robust gene expression from the ribosomal gene cluster (rDNA) are unclear. Here, we demonstrate that fission yeast cells facing glucose starvation assemble facultative heterochromatin in rDNA leading to its transcriptional repression. Glucose starvation induces quick dissociation of the ATF/CREB-family protein Atf1 from rDNA, where in turn the histone chaperone FACT is recruited to promote H3K9 methylation and heterochromatinization. We also identify the histone acetyltransferase Gcn5 as a repressor of rDNA heterochromatinization in glucose-rich conditions, and this protein dissociates from rDNA upon glucose starvation. Facultative heterochromatin formation in rDNA requires histone deacetylases Clr3 and both the RNAi-dependent and -independent gene silencing pathways. This is essential in adaptation to starvation since mutants lacking heterochromatin formation in rDNA lead to untimely cell death during glucose starvation.

## INTRODUCTION

The survival of living organisms is dependent on their ability to respond to environmental changes. This is usually achieved by swiftly modulating gene expression without inducing any genome rearrangements (1,2).

One such mechanism is epigenetic gene regulation, a process that has been extensively studied in eukaryotic cells. Changes in the environmental conditions may influence the chemical modification of histones and cytosine residues on DNA molecules without altering the sequence of DNA (3,4). These modifications are inheritable over multiple rounds of cell divisions and in some cases even by the offspring, but can be reversibly altered in response to environmental changes (5–7). The epigenetic control of gene expression is thus a viable strategy for quick and temporary adaptation to cellular stress.

Although fission yeast does not have constitutive DNA methylation (8), it shares many of epigenetic machineries that are ubiquitously found in other multicellular eukaryotes. In particular, studies using fission yeast identified the conserved mechanism of heterochromatinization via histone H3K9 methylation (9). In fission yeast, many of transcriptionally active regions (euchromatic regions) are enriched for histone H3 acetylated at K9, which is modified by histone acetyltransferases (HATs) such as Gcn5 (10,11). On the other hand, transcriptionally silenced regions such as peri-centromeres, sub-telomeres, and the mating-type region are characterized by constitutive heterochromatinization and histone methylation at the same H3K9 residue, which is established and maintained through the concerted actions of the histone methyltransferase Clr4 (human Suv39H1 homolog), the heterochromatin protein Swi6 (HP1 homolog), and the histone chaperone FACT (a conserved heterodimeric complex of SSRP1 and Spt16) (12–18). Importantly, heterochromatin in centromeric regions is formed by the collaboration of small interfering RNA, the RITS (RNA-induced transcriptional silencing) complex, and RDRC (RNA-directed RNA polymerase complex) (19–21).

The formation of heterochromatin has been shown to depend on the extracellular environment (facultative heterochromatinization). A well-known example of facultative heterochromatin is the inactivation of the X chromosome in female mammalian cells, which is established by histone methylation on H3K27 (22). Although fission yeast lacks H3K27 methylation, facultative heterochromatinization may occur by several mechanisms. For instance, H3K9 methylation can induce local facultative heterochromatinization at some meiosis-specific loci including *mei4*^+^ and *ssm4*^+^ during mitosis (13). These regions go by the name of heterochromatin islands, and are formed by the concerted action of RNA binding protein Mmi1 (involved in decay of meiosis-specific mRNA in mitosis) and the Red1-containing MTREC complex, independently of the RNAi machinery. Another mechanism utilizes the CCR4–NOT complex, involved in the stability control of mRNA poly-A tails, and the RNAi-dependent pathway, which are both crucial for the formation of heterochromatin domains (HOODs) seen in loci such as *mug5*^+^ and *mcp3*^+^ (23–27).

Along with epigenetic regulation, most eukaryotes control gene expression via stress-responsive ATF/CREB-type transcription factors, which are under the control of the SAPK (stress-activated protein kinase) signaling pathway (28). This stress-signaling unit regulates gene expression in response to various stimuli including heat, osmotic, and oxidative stress (29). In fission yeast, extracellular stress activates the Wis1 kinase, followed by phosphorylation of the Spc1/Sty1 kinase (homolog of budding yeast HOG1 and mammalian JNK and p38), which in turn induces the phosphorylation of Atf1, a fission yeast ATF/CREB-type transcription factor that is a homolog of ATF2 in mammalians (30–34). Phosphorylated Atf1 specifically binds short recognition sequences upstream of stress-responsive genes to activate transcription of downstream stress-responsive genes (29,35,36). In the promoter regions of some stress-responsive genes, Atf1 activates a positive feedback cycle resulting in enhanced transcriptional induction of upstream promotor-associated long non-coding transcripts by recruiting Gcn5 HAT and promoting H3K9 acetylation leading to local histone eviction (37). However, the mechanisms for gene repression in response to stress stimuli are much less understood.

In fission yeast, sudden depletion of glucose during exponential growth results in massive changes in the transcriptional landscape. In particular, the transcription of ribosome-related genes is markedly repressed, which, concomitant with global translational repression (38,39), shows that fission yeast cells temporarily slow down protein synthesis in response to starvation, most likely as an energy-saving mechanism. Several studies have indicated that heterochromatin marks and proteins contribute to rDNA silencing, and nitrogen starvation triggers heterochromatin formation, suggesting that rDNA transcription is also epigenetically silenced in response to glucose depletion (13,24,40–42). However, the detailed mechanisms are unclear.

In this study, we uncovered the molecular mechanisms of glucose starvation-induced gene silencing of rDNA via facultative heterochromatin formation. Importantly, mutants defective in rDNA heterochromatin formation dramatically lose viability under glucose-poor conditions, suggesting the pivotal role of rDNA heterochromatinization in the adaptation to starvation.

## MATERIALS AND METHODS

### Yeast strains, media and genetics

Fission yeast strains used in this study are listed in Supplementary Table S1. Strains used for chromatin immunoprecipitation (ChIP) and RT-PCR to detect RNA abundance were cultured as shown in our previous study (37). In brief, strains grown in the YES agar plate were incubated in YER liquid medium for 19 h at 30°C. To induce glucose starvation, cells in the log-phase were harvested in 50 ml tubes, washed twice in YED liquid medium containing 0.1% glucose, and then cultured in YED liquid medium at 30°C. The strain used for ChIP to assess the effect of high-temperature stress was cultured in YER liquid medium for 19 h at 30°C, followed by temperature shift up to 36°C. Cells were harvested at 15 min and 30 min after the temperature shift. For Western blotting, strains used in Figure 4C, 5H and Supplementary Figure S2C, S3A and S5H were incubated in YES liquid medium for 19 h at 30°C. Strains used in Supplementary Figure S2A were cultured in YER and then in YED liquid medium. To detect dead cells, 10^6^ cells were spotted on Phloxine B-containing YER and YED plates or cells were cultured in YED liquid medium containing Phloxine B.

Gene knock-in and knock-out were performed according to conventional transformation and genome integration methods (43,44). To construct the GFP-Swi6 strain, the *swi6*^+^ gene amplified from a wild type genome was inserted into the pFA-GFP-kan plasmid by using In-Fusion HD Cloning Kit (TaKaRa). From this plasmid, the *GFP-swi6-kan* fragment was amplified by PCR and introduced into the *swi6*Δ*::nat* strain. G418-resistance and nourseothricin-sensitive colonies were picked up, and then the proper GFP-Swi6 signal and normal growth were confirmed. Atf1 was overexpressed ectopically from chromosome II using a mutant *adh1* promoter, the *adh21* promoter. To insert the tev sequence into the *gcn5*^+^, the unstructured region of Gcn5 was selected by PHYRE2 Protein Fold Recognition Server (45). The *gcn5*^+^ or *ccr4*^+^ genes were inserted into the pFA-3HA-hph plasmid, and the tev sequence was inserted between Lys 148 and Met 149 of Gcn5 and between Leu 300 and Val 301 of Ccr4. From this plasmid, the *gcn5-tev-3HA-hph* or *ccr4-tev-3HA-hph* fragment was amplified by PCR and introduced into the *gcn5*Δ*::kan* or *ccr4*Δ*::kan* strain. Hygromycin-resistance and G418-sensitive colonies were picked up, and eventually the strains were properly selected by western blotting. To tag TEV protease with Gar2, the pADH-HBD-TEVp- ECFP plasmid was distributed (from Dr Masamitsu Sato) and the sequence of TEV protease was amplified and inserted between PacI and SacI sites in the pFA-kan plasmid to construct pFA-TEVp-kan plasmid. This plasmid was used as a template to tag the C-terminus of Gar2 with TEVp-kan.

### Chromatin immunoprecipitation (ChIP)

ChIP was performed as in our previous study (38) with some changes as described below. The cultures were incubated with formaldehyde (final 1%) for 15 min at 30°C, and then glycine (final 125 mM) was added and incubated at room temperature for an additional 5 min to stop the cross-linking reaction. Cells were washed twice with TBS and the cell pellets were frozen in liquid nitrogen. Cells were suspended in 200 μl of buffer I (50 mM HEPES/KOH (pH 7.5), 150 mM NaCl, 1 mM EDTA (pH 8.0), 1% Triton X-100, 0.1% sodium deoxycholate) with Complete protease inhibitor (Roche) and 1 mM phenylmethylsulfonyl fluoride and the cell membranes were disrupted using 0.5 mm zirconia beads and Multibead Shocker (Yasuikikai, Japan). Chromatin extracted from cells was fragmented into ∼500 bp fragment sizes on ice using Handy Sonic (UR-20P, Tomy Seiko, Japan; six repetitions of 30 sec sonication and 1 min interval, power 10), followed by centrifugation and collection of the supernatants.

For immunoprecipitations, Dynabeads Protein A (Invitrogen) was conjugated with α-Atf1 antibody (ab18123, abcam), α-H3K9me2 antibody (ab1220, abcam), α-H3K9me3 antibody (39161, Active motif), α-H3 antibody (ab1791, abcam), α-HA (12CA5, Sigma) antibody, α-GFP (632592, Clontech), α-myc (9E10, abcam), or α-Chp1 (ab18191, abcam) antibody for 3 h at 4°C, and Dynabeads Protein G (Invitrogen) was conjugated with either α-H3K9ac antibody (07–352, Millipore) or α-FLAG M2 antibody (F1804, Sigma) for 3 h at 4°C. The bead-antibody complex was mixed with the cell extracts for 3 h at 4°C. Thereafter, the beads were washed two times with chilled buffer I, lysis 500 buffer (50 mM HEPES/KOH (pH 7.5), 500 mM NaCl, 1 mM EDTA (pH 8.0) 1% TritonX-100, 0.1% sodium deoxycholate), wash buffer (10 mM Tris–HCl (pH 8.0), 1 mM EDTA (pH 8.0), 0.25 M LiCl, 0.5% NP-40, 0.5% sodium deoxycholate), and TE, respectively. 50 μl of elution buffer (20 mM Tris–HCl (pH 8.0), 100 mM NaCl, 20 mM EDTA (pH 8.0), 0.1% SDS) was added to the beads and heated for 15 min at 65°C, and the dissociated protein-DNA was collected from the beads. This manipulation was repeated twice and 100 μl of the solution was incubated overnight at 65°C. After that, 1 μl of proteinase K (20 mg/ml, Invitrogen) was added to the solution and incubated for 3 h at 55°C, followed by purification of DNA using FastGene Gel/PCR Extraction Kit (NIPPON Genetics). qPCR was performed by KAPA SYBR Fast qPCR kit (NIPPON Genetics) and StepOnePlus (Applied Biosystems) using the primer sets summarized in Supplementary Table S2.

### ChIP-seq library preparation

The DNA fragments of H3K9me2 were sheared to 300 bp using Covaris Focused-ultrasonicator S220 (Covaris). NGS libraries were prepared using NEBNext Ultra II DNA Library Prep Kit (NEB) and NEBNext Multiplex Oligos for Illumina (NEB). These libraries were deep sequenced by a commercial company (Macrogen) using Hiseq X (2 × 150 bp: Illumina).

### ChIP-seq data analysis

ChIP-seq data of Atf1 profiles from (37) were reanalyzed as follows. The regions with altered Atf1 binding were identified using callpeak and bdgdiff modules of macs2 (46). Callpeak options were set to -g 1.4e7, -q 0.01, –nomodel, and – extsize 147. We further set the options for bdgdiff to -g 60, -l 120, and –o -prefix. The same process was repeated for replicate 1 and replicate 2, and we extracted the common regions where the binding of Atf1 was reduced by intervene (47). Subsequently, bedtools (48) was used to extract the gene regions where Atf1 was reduced. The newly acquired reads derived from H3K9me2 were mapped to the *S. pombe* genome. To visualize the enrichment of Atf1 and H3K9me2, we generated bigwig files showing the log_2_ ratio (IP/input) using the bamcompare command of the deeptools package (49). In this case, the bin was set to 10. The visualization of bigwig files was done with the Integrative Genomics Viewer (50). For Atf1, representative data from replicate 2 are presented in this paper.

### Western blotting

The cells cultured as described above were centrifuged and washed twice with cold PBS followed by freezing using liquid nitrogen. The frozen cells were suspended with lysis buffer (50 mM Tris–HCl (pH 7.5), 1 mM EDTA (pH 8.0), 10% glycerol, 150 mM NaCl, 0.05% NP-40, 1 mM phenylmethylsulfonyl fluoride, 1 mM dithiothreitol, Complete protease inhibitor (Roche)) and cells were disrupted by 0.5 mm zirconia beads and Multibead Shocker (Yasuikikai, Japan). The supernatants were then collected by centrifugation and the protein concentration was measured. Equal amounts of SDS buffer were added to the solutions and heated at 100°C for 3 min. The resulting samples were resolved by standard SDS-PAGE on a 12% polyacrylamide gel and transferred to a PVDF membrane by Wet transfer system. α-Atf1 antibody (ab18123, abcam), α-HA antibody (12CA5, Sigma), and α-tubulin antibody (T6074, Sigma) were used as primary antibodies, and α-mouse IgG-HRP (GE Healthcare) and α-rat IgG-HRP (Santa Cruz) were used as the secondary antibody for protein detection.

### Cleavage of Gcn5-tev-HA and Ccr4-tev-HA by TEV protease *in vitro*

The cell extracts obtained by the procedure above were incubated with α-HA antibody (12CA5, Sigma) for 1.5 h at 4°C. This solution was then mixed with Dynabeads Protein A (Invitrogen), followed by incubation for 1.5 h at 4°C. Thereafter, the beads were washed 3 times with lysis buffer. 25 μl of TEV buffer (50 mM Tris–HCl (pH 8.0), 5 mM EDTA (pH 8.0), 1mM DTT) were added to the beads with or without adding 15 U TEV protease (T4455, Sigma) followed by overnight incubation at 30°C. α-HA antibody (12CA5, Sigma) was used as the primary antibody and α-mouse IgG-HRP (GE Healthcare) was used as the secondary antibody for protein detection.

### RNA purification and RT-PCR

**Figure 1.**
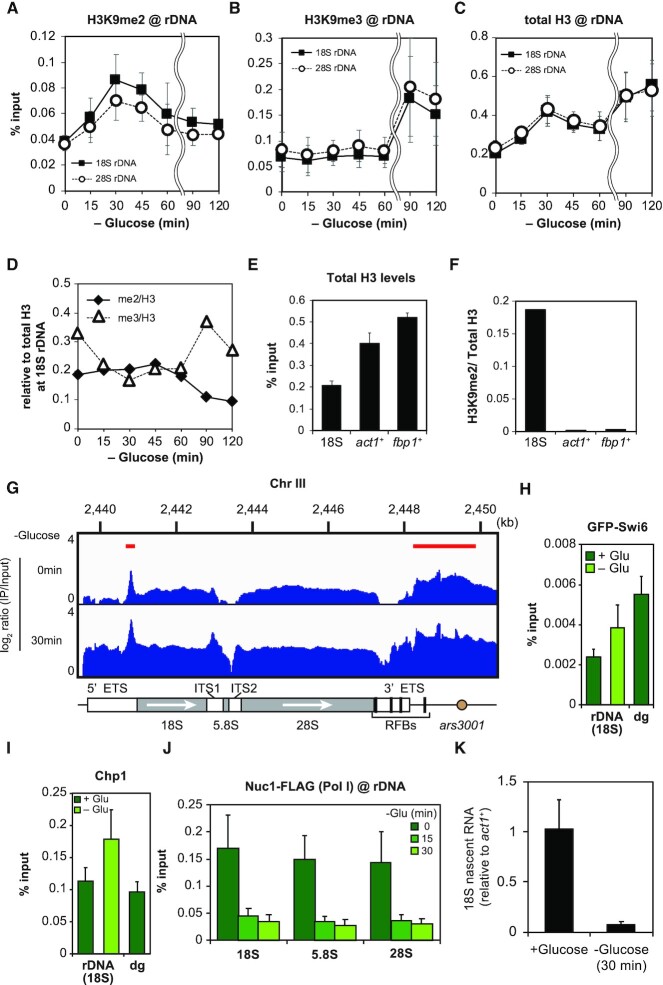
Glucose starvation increases H3K9 methylation in rDNA. (**A**) Time course of ChIP-qPCR experiments examining H3K9me2 levels in 18S (filled squares) and 28S rDNA (open circles) (mean ± SEM: *n* = 3). (**B**) Data of ChIP-qPCR showing the H3K9me3 levels in 18S (filled squares) and 28S rDNA (open circles) (mean ± SEM: *n* = 3). (**C**) ChIP-qPCR showing the total H3 levels in 18S (filled squares) and 28S rDNA (open circles) after glucose starvation (mean ± SEM: *n* = 3). (**D**) Graph showing the ratios of H3K9me2 (filled rhombus) and H3K9me3 (open triangles) relative to total H3. The average values of H3K9me2 and H3K9me3 in the three independent experiments are compared with that of the total H3 in the three independent experiments. Times indicate minutes after glucose starvation. (**E**) ChIP-qPCR data showing the amount of H3 in 18S rDNA, the *act1*^+^ gene, and the promoter of the *fbp1*^+^ gene in a glucose-rich medium (mean + SEM: *n* = 3). (**F**) Graph showing the ratio of H3K9me2 relative to total H3 in 18S rDNA, the *act1*^+^ gene and the promoter of the *fbp1*^+^ gene. The average values of H3K9me2 in the three independent experiments are compared with that of total H3 in the three independent experiments. (**G**) ChIP-seq profile of H3K9me2 in rDNA during glucose-rich (upper) and -poor conditions (lower). Red horizontal lines indicate the positions of regions with highly methylated H3K9 under glucose-rich conditions. The schematic diagram at the bottom indicates the rDNA annotated region. White arrows indicate the direction of transcription. The y-axis exhibits log_2_ ratio of IP/input (0–4). (**H** and **I**) Enrichment of HP1 and Chp1 in 18S rDNA and *dg* (peri-centromere). GFP-Swi6 (H) and Chp1 (I) are shown (mean + SEM: *n* = 6). Samples for glucose starvation were taken at 120 min. (**J**) ChIP-qPCR of Nuc1-FLAG, a component of RNA polymerase I, in each rDNA element. 0, 15, 30 min indicate the time in glucose-starved medium (mean + SEM: *n* = 3). (**K**) Graph showing the amount of newly synthesized (nascent) 18S rRNA before and after glucose starvation (mean + SD: *n* = 3). Nascent 18S rRNA levels were normalized with that of *act1*^+^ mRNA.

**Figure 2.**
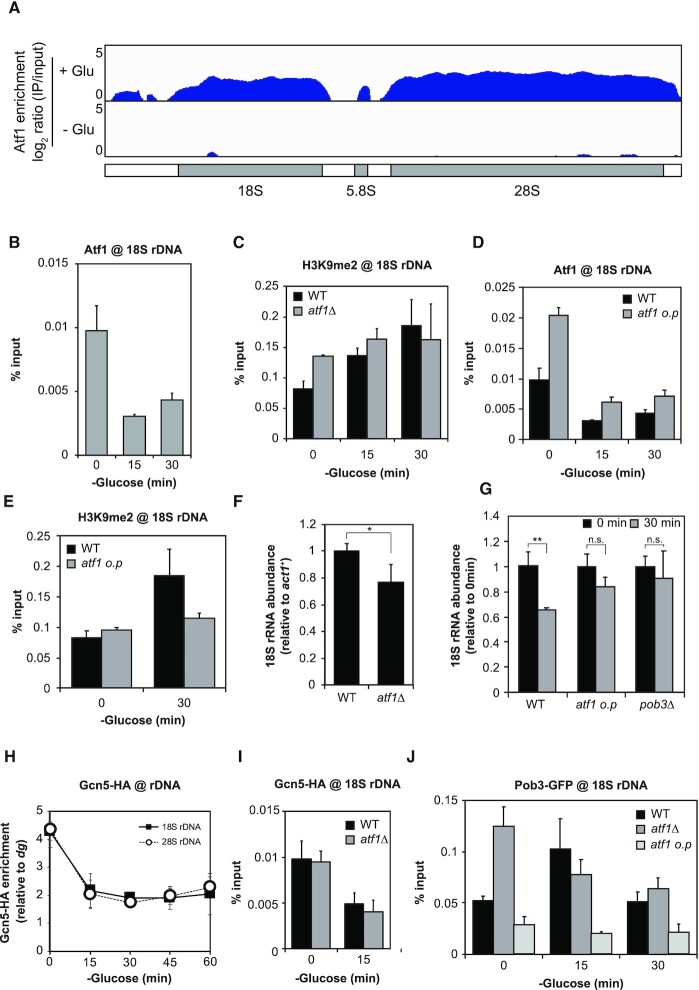
Dissociation of Atf1 from rDNA and accumulation of FACT for promotion of H3K9 methylation. (**A**) ChIP-seq data showing Atf1 enrichment in rDNA during glucose-rich (+Glu, 0 min, upper) and glucose-poor (–Glu, 15 min, lower) conditions. The schematic diagram below the plots indicates the annotated rDNA region. The y-axis exhibits log_2_ ratio of IP/input (0–5). (**B**) ChIP-qPCR results quantifying Atf1 occupancy in 18S rDNA during the early course of glucose starvation (mean + SEM: *n* = 3). (**C**) H3K9me2 levels in 18S rDNA upon glucose starvation in the wild type (WT) and *atf1*Δ strains (mean + SEM: *n* = 3). (**D**) ChIP-qPCR quantifying Atf1 occupancy in 18S rDNA in the wild type (WT) and the strain overexpressing Atf1 (*atf1 o.p*) (mean + SEM: *n* = 3). The wild type control data is the same as in (B). (**E**) H3K9me2 levels in 18S rDNA under glucose-rich (0 min) and low glucose (30 min) conditions in the wild type (WT) and *atf1 o.p* strains (mean + SEM: *n* = 3). The wild type data is identical to that in (C). (**F** and **G**) 18S rRNA abundance relative to *act1*^+^ mRNA in the wild type (WT) and *atf1*Δ strains under glucose-rich condition (**F**) and in the wild type (WT), atf1 overexpression strain (*atf1 o.p*) and *pob3*Δ strains during glucose starvation (**G**). Student's *t*-test (two-tailed) was applied and statistical significance was indicated (*P < 0.05, **P < 0.01, n.s., not significant), (mean + SD, *n* = 3). (**H**) ChIP-qPCR showing the localization of Gcn5-HA in 18S (filled squares) and 28S rDNA (open circles) after glucose starvation. The % input of Gcn5-HA was normalized to the % input of the *dg* region (mean ± SEM: *n* = 4). (**I**) Enrichment of Gcn5–HA in 18S rDNA in the wild type (WT) and *atf1*Δ strains (mean + SEM: *n* = 3). (**J**) Binding of Pob3-GFP to 18S rDNA in the wild type (WT), *atf1*Δ and *atf1 o.p* strains. Times after glucose depletion are indicated below the graph (mean + SEM: *n* = 3).

**Figure 3.**
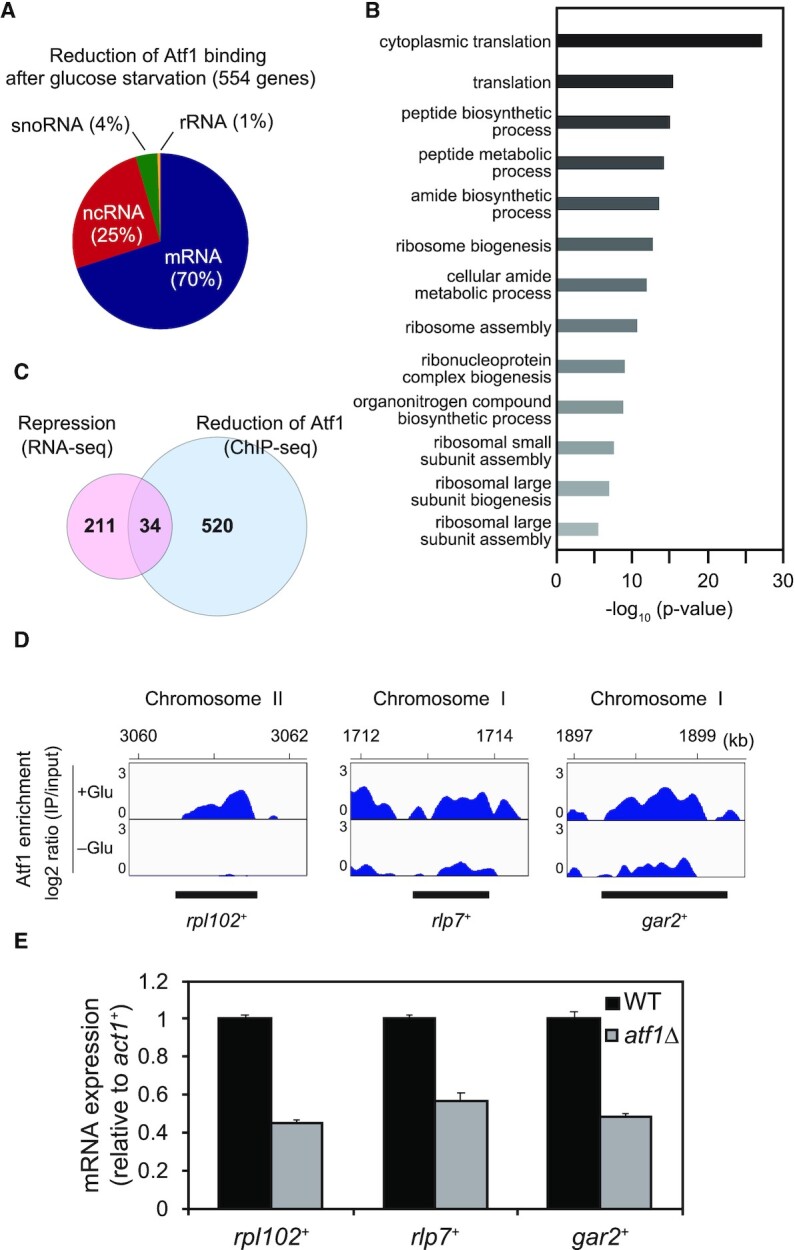
Genome-wide correlation between Atf1-dissociation sites and transcriptional silencing of ribosome-related genes during glucose starvation. (**A**) A pie chart showing the proportion of gene types marked by glucose starvation-induced Atf1 dissociation. The percentage of mRNA, ncRNA, snoRNA, and rRNA in 554 genes are shown in parentheses. (**B**) Graph showing the enrichment of each gene ontology (GO) term. The GO terms were identified using Generic Gene Ontology Term Finder. The p-value cutoff was set to 0.01. (**C**) Venn-diagram showing the number of genes overlapping between repressed transcripts and genes exhibiting Atf1 dissociation. (**D**) ChIP-seq data of Atf1 for representative loci in the overlapped region shown in (**C**). ‘+Glu’ and ‘–Glu’ represent data at 0min and 15 min of glucose depletion. The y-axis exhibits log_2_ ratio of IP/input (0–3). The numbers at the top indicate coordinates on the corresponding chromosomes (kb) and the bottom black lines indicate positions of gene regions. (**E**) mRNA expression levels of *rpl102*^+^, *rlp7*^+^ and *gar2*^+^ relative to that of the *act1*^+^ gene in the wild type (WT) and *atf1*Δ cells (mean + SD: *n* = 3).

In Figures 2F, G, 3E, Supplementary Figure S5A and B, RNA extraction was performed according to the previous method we have shown (39). In Figure 1K, nascent RNA was detected by the following method based on the previous protocol (51). First, 250 μl of 4-thiouridine (7.5 mg/ml, 13957–31-8, Cayman Chemical) were added to 50 mL of YER and YED culture medium. The treated cells were shaken for 10 min, harvested, and frozen at -80°C. Then, RNA was extracted according to the method described above. Extracted RNA was incubated with biotin buffer (10 mM Tris–HCl (pH 7.4), 1 mM EDTA, 0.4 mg EZ-link HPDP-Biotin (21341, Thermo Scientific)) for 90 min at room temperature. The biotinylation solutions were mixed with chloroform: isoamyl alcohol. After centrifugation, upper phases were mixed with 1 volume of isopropanol and 1/10 volume of 5 M NaCl, followed by overnight incubation at -80°C. The supernatant after centrifugation was discarded and 500 μl of 75% ethanol were added. After removing ethanol completely, the pellet was resuspended in RNase-free water. Before mixing the biotinylated RNA with Dynabeads M-280 Streptavidin (Invitrogen), the beads were washed three times with MPG buffer (100 mM Tris–HCl (pH 7.4), 1M NaCl, 10 mM EDTA). The beads in MPG buffer were then incubated with Yeast tRNA (AM7119, Invitrogen) for 30 min at room temperature. Lastly, the beads were washed three times with MPG buffer. Biotinylated RNA, heated at 65°C for 5 min, then cooled at room temperature for 1 min, was added to MPG buffer containing equilibrated streptavidin beads. After 40 min of incubation at room temperature, beads were washed three times with MPG buffer preheated to 65°C. To elute RNA, the beads were incubated with 5% β-mercaptoethanol for 5 min at room temperature, followed by collecting solutions. This step was repeated twice. RNA was further purified from the pooled β-mercaptoethanol-containing elution using RNAqueous-Micro Total RNA isolation Kit (AM1931, Invitrogen) according to the following procedure. The elution was mixed with lysis solution and ethanol, followed by applying the mixture onto columns. Then, columns were washed three times with wash solution 1 and washed twice with wash solution 2/3. RNA was finally eluted by RNase-free water preheated at 85°C. The prepared RNA was reverse transcribed using PrimeScript™ RT reagent Kit with gDNA Eraser (RR047A, TaKaRa) according to the manufacturer's instructions. The resulting cDNA was used as a template for qPCR following the methods described in the ChIP section. Primers in this study were listed in Supplementary Table S2.

### Microscopy

For observation of Clr6-GFP, Sir2-GFP, cells were fixed with formaldehyde (final 3.2%) for 15 min at room temperature. For observation of Hst2-GFP, cells were fixed with 50% ethanol for 20 min at room temperature. Cells were washed with PBS and stained by DAPI. Imaging was conducted with EVOS FL Cell Imaging System (Thermo Fisher Scientific) equipped with UPlanSApo 100×/1.40 oil objective lens (Olympus). The signal at 530 nm wavelength was measured to detect the dead cells stained by Phloxine B.

### Measurement of cell viability

Each strain from fresh YES agar plates was cultured in YED liquid medium for 24 h at 30°C. Cells were diluted to the proper concentration and then 300 cells were seeded into YES agar plates. Cells were diluted to a concentration of 10^6^ cells/ml every 24 h to measure cell viability for a long period of time.

### Measurement of cell growth

To monitor the density of cells cultured in YER and YED over time, we used ODBox-C/ODMonitor systems (TAITEC, Japan). Glass test tubes containing cell culture were tilted at 30° and OD_950_ was measured every minute. The measured OD950 was automatically converted to OD_600_.

## RESULTS

### Nutritional starvation increases H3K9 methylation in rDNA

Ribosomal RNAs (rRNAs) are quite abundant, occupying up to 60% of all transcripts in vegetatively growing yeast cells (52). Our previous study demonstrated that the transcription of ribosome-related genes including rDNA, along with that of various genes required for growth, is markedly repressed in the early response to glucose starvation (38). We therefore investigated the mechanisms of transcriptional repression of rDNA during glucose starvation.

A well-known mechanism for gene silencing is the formation of heterochromatin caused by methylation at lysine 9 of histone H3 (H3K9). We thus conducted chromatin immunoprecipitation (ChIP) experiments to monitor H3K9 methylation levels at fission yeast rDNA during glucose starvation. We found that dimethylation of H3K9 (H3K9me2) in 18S and 28S rDNA was increased about 2-fold after 30 min in the glucose-poor medium. Afterward, H3K9me2 progressively decreased to the basal level under glucose-rich conditions (Figure 1A). We further investigated trimethylation of H3K9 (H3K9me3), which often appears after the appearance of H3K9me2. Though the H3K9me3 level remained almost unchanged until 60 min, as expected, it markedly increased at 90 and 120 min (Figure 1B).

We also examined the total H3 occupancy in rDNA (Figure 1C). The increase of H3K9me2 and H3K9me3 appeared to be associated with the increase of the total H3 occupancy in rDNA at the early (0–30 min) and late (60–120 min) stages of adaptation to glucose starvation, respectively (Figure 1A–C). When we plotted a ratio of H3K9me2 or H3K9me3 relative to the total H3 occupancy (H3K9me2/H3 or H3K9me3/H3, respectively, Figure 1D), during 0–60 min of glucose starvation, the H3K9me2/H3 ratio was almost constant. After 60 min, it gradually decreased, coupled with an increase in the relative enrichment of H3K9me3. The H3K9me3/H3 was reduced by about 40% during 15–60 min of glucose starvation but restored after 60 min.

It should be noted that the density of H3 in rDNA was much smaller than those in *act1*^+^ and the promoter of the *fbp1*^+^ (Figure 1E), while the H3K9me2/H3 ratio was strikingly higher in rDNA (Figure 1F). It is therefore likely that some nucleosomes containing methylated H3K9 are accumulated at certain locations in each rDNA repeat even under nutritious conditions, and newly recruited histone H3 to each rDNA repeat is gradually methylated to form heterochromatin after glucose starvation (see a schematic diagram of Supplementary Figure S1A).

Therefore, we next investigated the localization of methylated histones under both glucose-rich and -poor conditions. We designed primer sets at five locations throughout the rDNA and performed qPCR, and found that histones containing H3K9me2 are relatively enriched in the 5′ region of *ars3001* (primer 1 in Supplementary Figure S1B) and 5′ ETS (external transcribed spacer) (primer 3). Upon glucose starvation, the accumulation of H3K9me2 occurred over the entire rDNA region (Supplementary Figure S1B). We also conducted ChIP-seq to study more precise H3K9me2 distribution (Figure 1G). H3K9me2 was enriched at a peak in the 5′ ETS region and in the 5′ region of *ars3001* in glucose-rich conditions (red horizontal bars in the upper panel). In glucose-starved conditions, the occupancy of H3K9me2 increased over the entire rDNA region, consistent with the ChIP-qPCR results.

The binding of HP1 to methylated H3K9 is required for the assembly of mature heterochromatin (9). Fission yeast has two HP1 homologs, Swi6 and Chp2, with Swi6 playing a particularly essential role in heterochromatinization (40,53). We thus conducted ChIP experiments targeting GFP-N-tagged Swi6 (GFP-Swi6) (Figure 1H). Under glucose-rich conditions, the localization of GFP-Swi6 to rDNA was about 45% of the peri-centromeres (*dg*), but glucose starvation increased the amount of GFP-Swi6 to about 70% of that in peri-centromeres. We also observed that chromodomain protein Chp1, which contributes to heterochromatin formation (54,55), was enriched 1.6-fold upon glucose starvation (Figure 1I).

In budding yeast, it has been reported that oxidative stress induces dissociation of RNA polymerase I in rDNA coupled with a decrease in nascent rRNA (56). We then examined whether the facultative heterochromatin in rDNA indeed prevents rRNA synthesis. First, we monitored the enrichment of Nuc1, a component of RNA polymerase I, in rDNA by ChIP experiments. As expected, Nuc1-FLAG levels in the 18S, 5.8S and 28S rDNA regions were drastically reduced after glucose starvation (Figure 1J). We further investigated the levels of nascent rRNA by measuring biotinylated RNA captured with streptavidin beads as described previously (51). As expected, nascent 18S rRNA was strikingly decreased after glucose starvation (Figure 1K). These results suggest that transcriptional silencing indeed occurs in rDNA during glucose starvation. We also examined H3K9me2 levels in other well-described constitutive heterochromatic regions such as the peri-centromeres (*dg*), *mat* locus, and sub-telomeres. They all exhibited no changes in H3K9me2 levels in glucose-starved cells (Supplementary Figure S1C–E). Additionally, we studied H3K9me2 levels in the constitutively transcribed locus of *act1*^+^, and found that *act1*^+^ also exhibited a slight increase in H3K9me2 levels upon glucose starvation (Supplementary Figure S1F, left). This result is consistent with the previous observation that >200 euchromatin regions, including the *act1*^+^ gene, are individually silenced by RNAi-dependent facultative heterochromatin formation in cell quiescence (41). On the other hand, we detected little changes in H3K9me2 levels in the promoter region of *fbp1*^+^ (fructose-1,6-bisphosphatase), which is markedly activated in response to glucose starvation (Supplementary Figure S1F, right).

We further examined whether the rDNA heterochromatinization occurs in response to different types of stress such as high-temperature shift, and found that H3K9me2 was elevated in rDNA soon after the high-temperature shift to 36°C (Supplementary Figure S1G), suggesting that other stresses can induce rDNA heterochromatinization.

### Replacement of Atf1 by FACT leads to heterochromatinization in rDNA

The mechanism of facultative heterochromatinization in rDNA during glucose starvation is barely understood. A fission yeast ATF/CREB family transcription factor Atf1 regulates the expression of stress-responsive genes in response to environmental changes (29,38) and is involved in *mat* locus silencing (57,58). For instance, upon glucose starvation, Atf1 and Gcn5 HAT are both recruited to the upstream region of the *fbp1*^+^ gene and promote histone H3K9 acetylation (37).

Considering the previous studies described above, we speculated that the alteration of Atf1 localization contributes to the rDNA heterochromatinization. We therefore reanalyzed the ChIP-seq data of Atf1 (Figure 2A and B). We found that Atf1 accumulated in rDNA during glucose-rich conditions but the Atf1 binding was strikingly reduced in glucose-poor conditions, although total Atf1 protein abundance in the cell remained constant (Supplementary Figure S2A). Such a dissociation of Atf1 from rDNA was not regulated by the stress-activated kinase Spc1/Sty1, since Atf1 could immediately dissociate from rDNA even in *spc1*Δ (Supplementary Figure S2B). Furthermore, we found that the deletion of *atf1*^+^ (*atf1*Δ) caused rDNA to exhibit consistently elevated levels of H3K9me2 irrespective of extracellular glucose concentration (Figure 2C), indicating that Atf1 may be a critical player in the starvation-dependent alteration of H3K9 methylation.

This idea led us to predict that an excessive accumulation of Atf1 in rDNA would inhibit the starvation-induced methylation of H3K9. We thus constructed a strain that constitutively overexpresses Atf1 from the Z locus (adjacent to the *zfs1*^+^ gene on chromosome II) under the control of an *adh21* promoter (59). Atf1 was overexpressed by about 1.7-fold in this strain (*atf1 o.p*) compared to the wild type (Supplementary Figure S2C). We found that the overexpressed Atf1 was enriched in rDNA about twice as much as in the wild type in glucose-rich conditions (Figure 2D). Atf1 was retained in the rDNA region even during glucose starvation at comparable levels to that in the wild type cells in a glucose-rich medium (Figure 2D). More importantly, Atf1 overproduction inhibited the increase in H3K9me2 levels of rDNA even after 30 min of glucose starvation (Figure 2E). These results support the hypothesis that Atf1 inhibits the accumulation of H3K9 methylation in rDNA.

We next investigated the 18S rRNA abundance in *atf1*Δ and *atf1 o.p* strains by RT-qPCR (Figure 2F and G). We found that the *atf1* deletion reduced 18S rRNA abundance under glucose-rich conditions (Figure 2F), consistent with the above-mentioned hypothesis. On the other hand, in the *atf1 o.p* strain, the 18S rRNA abundance increased to about twice that of the wild type in a glucose-rich medium. (Supplementary Figure S2D). It should be noted that the 18S rRNA abundance in the *atf1 o.p* strain stayed at relatively high levels during glucose starvation (Figure 2G). These results again support the notion that the Atf1-binding to rDNA inhibits the rDNA heterochromatinization, and the Atf1-dissociation from rDNA upon glucose starvation triggers the induction of H3K9 methylation, leading to repression of rRNA expression.

Next, we examined the involvement of Gcn5, which functions cooperatively with Atf1. ChIP experiments revealed that Gcn5 specifically accumulated in rDNA but not in *prp3*^+^ or peri-centromeric (*dg*) regions in the nutritious condition (Supplementary Figure S2E). More than half of Gcn5 was immediately dissociated from rDNA due to glucose depletion (Figure 2H). In addition, deletion of *atf1*^+^ did not affect Gcn5 occupancy on rDNA (Figure 2I). These results indicate that Gcn5 is displaced from the rDNA independently of Atf1. Therefore, it is likely that Atf1 controls H3K9 methylation in rDNA via factors other than Gcn5.

We thus explored the role of the histone chaperone FACT (facilitates chromatin transcription). FACT is a heterodimer consisting of two proteins, Spt16 and Pob3, and is reported to maintain the epigenetic stability of heterochromatin by preventing histone turnover (14,16). We hypothesized that FACT is recruited to the rDNA region in response to glucose starvation, and then prevents the turnover of methylated H3, consequently promoting rDNA facultative heterochromatinization.

ChIP experiments revealed that GFP-tagged Pob3 transiently accumulated to rDNA upon glucose depletion (Figure 2J, black bars). We also found an excessive accumulation of Pob3 in *atf1*Δ in glucose-rich conditions (Figure 2J, gray bars) and lower accumulation of Pob3 in the *atf1 o.p* strain irrespective of nutritional conditions (Figure 2J, light gray bars), suggesting that Atf1 prevents the Pob3 recruitment to rDNA.

We further examined H3K9me2 levels in *pob3*Δ by ChIP-qPCR and found that they were indeed reduced to the basal level (Supplementary Figure S2F). We also observed that 18S rRNA abundance in *pob3*Δ increased to about 1.5-fold that in the wild type under glucose-rich conditions (Supplementary Figure S2D), and stayed at high levels during glucose starvation (Figure 2G). Taken together, these results suggest that Atf1 excludes FACT from the rDNA region in glucose-rich conditions, leading to a faster turnover of methylated histone H3 and consequently to the repression of heterochromatinization in rDNA. After dissociation of Atf1 from rDNA upon glucose starvation, it is likely that FACT is recruited to rDNA and increases methylated histone H3 levels in rDNA.

FACT is known to assist the reassembly of nucleosomes after the passage of RNAPII and along with another histone chaperone, suppressor of Ty 6 (Spt6) (60,61), contributes to the recycling of histone H3 during transcription (62,63). It should be noted that Spt6 also contributes to the rDNA silencing (64). Therefore, it is plausible that Spt6 is also involved in maintaining methylated histone H3 and rDNA heterochromatinization. As expected, we found that the deletion of *spt6*^+^ resulted in a significant reduction of H3K9me2 levels in rDNA (Supplementary Figure S2F). However, unlike FACT, Spt6 itself was not markedly enriched in rDNA during glucose starvation (Supplementary Figure S2G), suggesting that the high levels of H3K9 methylation in rDNA under glucose-starved conditions are mainly maintained by FACT.

### Dissociation of Atf1 during glucose starvation facilitates transcriptional repression of ribosome-related genes

As demonstrated above, heterochromatinization and transcriptional repression were facilitated by the dissociation of Atf1 from rDNA. To search for genomic regions that share this mechanism, we investigated the genome-wide distribution of Atf1 before and after glucose depletion. We reanalyzed our previous data (37), focusing on genomic regions where Atf1 enrichment was reduced during glucose starvation. We found that Atf1 dissociated from 554 genes (including 388 mRNAs, 141 ncRNAs, 22 snoRNAs and 3 rRNAs) after glucose depletion (Figure 3A, Supplementary Table S3). This number of Atf1-dissociating regions was more than that reported in the case of oxidative stress response (65).

To extract a common functional role for those regions, we performed gene ontology (GO) analysis using Generic Gene Ontology Term Finder (66,67). The GO analysis revealed that many Atf1-dissociating regions are correlated with genes for translation, ribosome biogenesis, and ribosome assembly (Figure 3B, Supplementary Table S4). These results suggest that transcription of some ribosome-related genes could be repressed by the dissociation of Atf1 in response to glucose depletion. The functions of the starvation-repressed ncRNAs are not known, but they might also contribute to ribosome biogenesis.

Our previous analysis demonstrated that glucose starvation quickly represses the transcription of 245 mRNAs (38). To visualize the relationship between these starvation-repressed mRNAs and the Atf1-dissociating regions, we prepared Venn diagrams to visualize the number of genes exhibiting both transcriptional repression and Atf1 dissociation upon glucose depletion. As shown in Figure 3C and Supplementary Table S5, 34 genes exhibited both transcriptional repression and Atf1 dissociation. Among these, eight were ribosome-related genes including *rpl102*^+^, *rlp7*^+^ and *gar2*^+^. Figure 3D indicates the Atf1 ChIP-seq results for the *rpl102*^+^, *rlp7*^+^ and *gar2*^+^ loci, showing the extent of Atf1 depletion at these loci during glucose starvation. To investigate whether Atf1 dissociation from these regions represses their transcription, we performed RT-qPCR in the wild type and *atf1*Δ strain. We found that the expression of these genes was markedly reduced in *atf1*Δ as compared to the wild type (Figure 3E). These results demonstrate that the Atf1-mediated transcriptional repression mechanism may also function in some ribosome-related genes.

### Local inactivation of Gcn5 in rDNA leads to heterochromatinization

**Figure 4.**
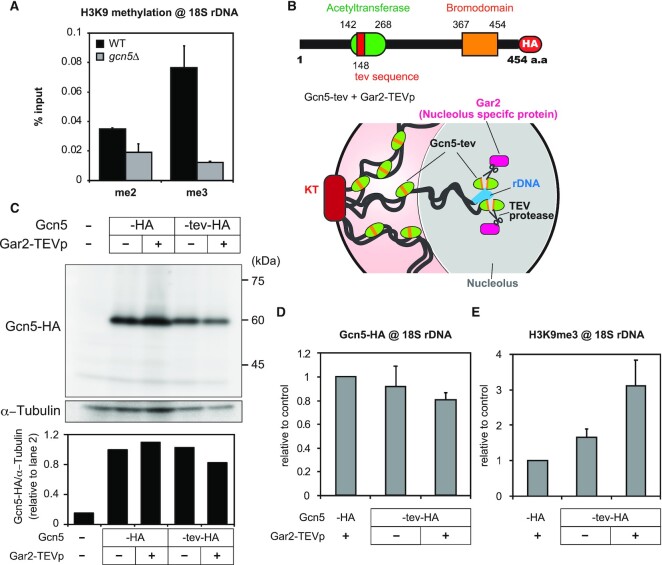
Targeted inactivation of Gcn5 in rDNA leads to an increase in H3K9 methylation. (**A**) H3K9me2 and H3K9me3 levels in 18S rDNA in the wild type (WT) and *gcn5*Δ strains under glucose-rich conditions (mean + SEM: *n* = 3). (**B**) A schematic diagram of Gcn5 domain structure. (top) The tev sequence is inserted to the HAT domain (red segment). (bottom) A diagram showing selective TEV-mediated cleavage of Gcn5 in rDNA. Nucleolus-specific protein Gar2, tagged with TEV protease cleaves Gcn5-tev only in the nucleolus. (**C**) (top) Immunoblotting of Gcn5-HA (–HA), Gcn5-tev-HA (-tev-HA) and α−Tubulin, in the presence (+) or absence (–) of Gar2-TEVp expression. Gcn5 (–) indicates control data using the wild type with non-tagged Gcn5. (bottom) The bands of Gcn5-HA were quantified and normalized to those of α−Tubulin. (**D** and **E**) Bar graph showing the data of ChIP-qPCR experiments for Gcn5-HA enrichment (**D**) and H3K9me3 levels (**E**) in 18S rDNA of indicated strains (mean + SEM: *n* = 3).

As shown in Figure 2H, Gcn5 HAT dissociated from rDNA in response to glucose starvation. Since Gcn5 is a histone acetyltransferase, we predicted that the dissociation of Gcn5 from rDNA would trigger the heterochromatin formation. Therefore, it was assumed that H3K9 methylation would increase in the *gcn5*Δ strain even under nutritious conditions. However, unexpectedly, methylation of H3K9 in *gcn5*Δ was reduced than in the wild type (Figure 4A). Previous observations revealed that the loss of Gcn5 weakens sub-telomeric silencing (68). These repressive roles of Gcn5 seem contradictory to its widely believed function in transcriptional activation, though Gcn5 is reportedly involved in the silencing of the *ste11*^+^ gene (master regulator of sexual differentiation) (69).

Because disruption of *gcn5* causes global transcriptional changes (69–71), indirect effects of Gcn5 deletion might mask its local or specific functions in rDNA. In order to assess the true contribution of Gcn5 in rDNA, we attempted to induce local degradation of Gcn5 specifically within rDNA by rDNA-tethered TEV (Tobacco Etch Virus) protease which recognizes and cleaves the tev sequence ENLYFQ*S (* is the cleavage site) (72). We generated a strain that harbors the *gcn5-tev-HA* gene with the tev sequence insertion in the HAT domain of Gcn5. We confirmed that TEV protease indeed cleaved the target sequence of Gcn5-tev-HA *in vitro* (Supplementary Figure S3A). TEV protease was then tagged with the C-terminus of Gar2 (Gar2-TEVp). Since Gar2 is known to reside only in the nucleolus (73) (Figure 4B), Gar2-TEVp is expected to specifically degrade Gcn5-tev-HA associated with rDNA in the nucleolus.

We first confirmed that Gcn5-tev-HA is expressed to the same extent as Gcn5-HA (Figure 4C). When Gar2-TEVp was co-expressed, the entire Gcn5-tev-HA abundance was slightly reduced (Figure 4C). No degraded fragments of Gcn5-tev-HA were detected, suggesting that the amount of Gcn5 bound to rDNA is a small fraction of the total Gcn5 (Figure 4C). It should be noted that HA-mediated ChIP experiments exhibited a slight but significant reduction in rDNA-bound Gcn5-tev-HA when Gar2-TEVp was expressed (Figure 4D). We think Gcn5-tev-HA in rDNA was indeed cleaved by Gar2-TEVp from the following observations. When we tagged GFP to the N-terminus of Gcn5 (at the expense of function) and measured its accumulation in rDNA, its amount decreased to about 60% of that in the control strain (Supplementary Figure S3B). Therefore, we consider that the abundant binding of Gcn5-tev-HA in rDNA in the presence of Gar2-TEVp may be explained by the post-cleavage retention of the C-terminus inactive remnant of Gcn5-tev-HA in rDNA, since the chromatin-binding bromodomain is located in the C-terminus of Gcn5 (10) (see the upper diagram in Figure 4B).

More importantly, H3K9me3 levels in rDNA under glucose-rich conditions were notably increased when Gcn5-tev-HA and Gar2-TEVp were co-expressed (Figure 4E). On the other hand, H3K9me3 levels at other loci (*abp1*^+^ and *obr1*^+^) known to be influenced by Gcn5 (69) were not significantly affected (Supplementary Figure S3C). Since the N-terminal-truncated Gcn5 without a HAT domain does not have HAT activity, even though it may remain in the rDNA region, it is not likely to inhibit the enrichment of methylated H3K9 in rDNA. All these results can be explained by the notion that H3K9 methylation in rDNA is repressed by Gcn5 under glucose-rich conditions but derepressed after dissociation of Gcn5 from rDNA in response to glucose depletion.

### H3K14 deacetylase Clr3 is involved in the control of H3K9 methylation in rDNA

We demonstrated that glucose starvation led to the dissociation of Gcn5 and the recruitment of FACT in rDNA, both of which contributed to the induction of higher levels of H3K9 methylation. Considering the process of H3K9 methylation, acetylation of H3K9 should be removed before histone methylation. We thus explored the involvement of H3K9 histone deacetylases (HDACs) in rDNA histone methylation.

The deacetylation of acetylated H3K9 (H3K9ac) is mediated by NAD-dependent deacetylases from the Sirtuin family (Sir2 and Hst2 in *S. pombe*) and Clr6, a fission yeast homolog of human HDAC1 (74–76). We first examined the localization of these proteins. Although it has been demonstrated that Clr6 co-localizes exclusively to DAPI-stained chromosomes (74), our observation showed that it also weakly associated with the nucleolus (Supplementary Figure S4A). Moreover, Sir2-GFP localized to the entire nucleus, as previously reported, whereas little nuclear localization of Hst2-GFP was observed, unlike previous observations (75,77) (Supplementary Figure S4A). Consistent with these results, Clr6-GFP and Sir2-GFP, but not Hst2-GFP, were enriched in 18S rDNA region (Supplementary Figure S4B).

We then examined H3K9ac levels of rDNA in the *clr6-1*, *sir2*Δ, and *hst2*Δ mutants. Although it had been shown by reporter gene assay that Sir2 and Hst2 contribute to the silencing in rDNA (75), H3K9ac levels did not increase in any of these mutants compared to the wild type (Supplementary Figure S4C). Crucially, glucose starvation did not attenuate the rDNA H3K9ac levels in the wild type (Supplementary Figure S4C). These results suggest that the deacetylation of H3K9ac by these HDACs is dispensable for the H3K9 methylation in rDNA.

On the other hand, several studies have indicated that the deacetylation of H3K14 via Clr3 HDAC and subsequent ubiquitination of H3K14 promotes H3K9 methylation via Clr4 (17,74,78–80). Consistent with these observations, H3K9me2 was completely abolished in 18S rDNA in the *clr3*Δ mutant (Supplementary Figure S4D). This result indicates that deacetylation of H3K14 by Clr3 is involved in the H3K9 methylation in rDNA.

### Both the RNAi-dependent and RNAi-independent pathways are required for H3K9 methylation in rDNA

In fission yeast, the establishment and maintenance of constitutive heterochromatic regions such as peri-centromeres require RNA interference (RNAi) to recruit the histone methyltransferase Clr4 (13,21,81). It should be noted that the RNAi component Ago1 is also associated with rDNA regions (24). We thus predicted that the RNAi-dependent pathway is involved in H3K9 methylation in rDNA.

**Figure 5.**
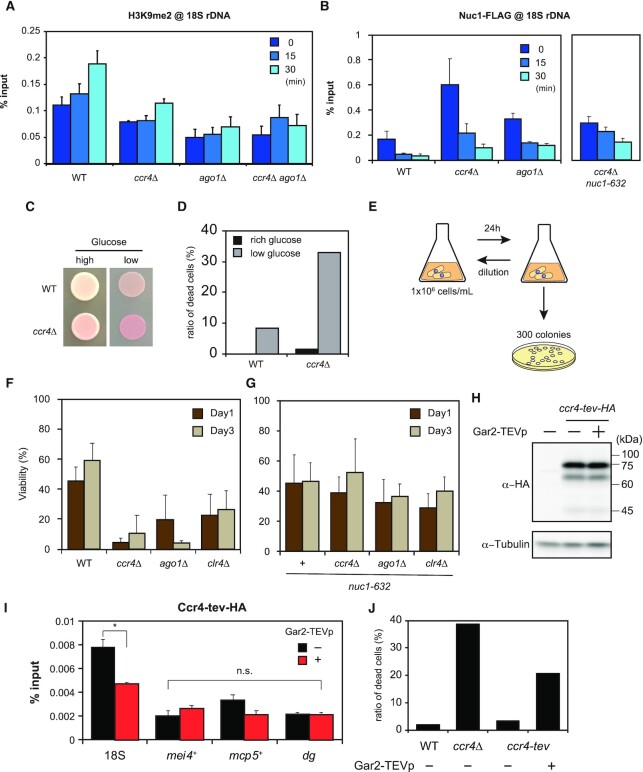
RNAi-dependent and RNAi-independent H3K9 methylation in rDNA during glucose starvation and crucial roles in cellular survival. (**A**) ChIP-qPCR data showing H3K9 methylation levels in 18S rDNA in the wild type (WT, *n* = 4), *ccr4*Δ (*n* = 3), *ago1*Δ (*n* = 3) and *ccr4*Δ *ago1*Δ (*n* = 3) mutants. Time indicates minutes after glucose depletion (mean + SEM). (**B**) ChIP-qPCR results of Nuc1-FLAG (RNA polymerase I) in 18S rDNA in the wild type (WT, *n* = 3), *ccr4*Δ (*n* = 5), *ago1*Δ (*n* = 3) and *ccr4*Δ *nuc1-632* (*n* = 3) strains. (mean + SEM). The data for the wild type is identical to that in Figure 1J. (**C**) Spot tests showing viability of the wild type (WT) and *ccr4*Δ on glucose-rich (high) and glucose-poor (low) agar media. The dead cells form darker red-colored colonies on agar media containing Phloxine B. (**D**) The percentage of dead cells in the wild type (WT) and *ccr4*Δ cultures estimated by microscopic observation of Phloxine B treated cells at 530 nm wavelength. Data for low glucose was taken at 24 h of starvation (*n* > 180 cells). (**E**) A diagram of cell viability assay. Cells were cultured in glucose-starved medium for 24 h, and 300 colonies were inoculated on the YES agar plates. Viable colonies were counted. To use cells at the same cell density (1 × 10^6^ cells/ml), cells were diluted and cultured in glucose-starved liquid media every 24 h. (**F**) Viability data of (E) during glucose starvation in the wild type (WT), *ccr4*Δ, *ago1*Δ and *clr4*Δ strains (mean + SD: *n* = 3). (**G**) Viability data of (E) during glucose starvation in *nuc1-632*, *nuc1-632 ccr4*Δ, *nuc1-632 ago1*Δ and *nuc1-632 clr4*Δ strains (mean + SD: *n* = 4). (**H**) Immunoblotting of Ccr4-HA with tev sequence (Ccr4-tev-HA). We could not detect the cleaved fragments of bulk of Ccr4-tev-HA even when Gar2-TEVp was co-expressed. (**I**) ChIP-qPCR showing that the occupancy of Ccr4-tev-HA was decreased in 18S rDNA but not in *mei4*^+^, *mcp5*^+^ and *dg* regions, when TEV protease was co-expressed (mean + SEM: *n* = 3). Welch's *t*-test (two-tailed) was applied and statistical significance was indicated (*P< 0.05, n.s., not significant). (**J**) Percentage of dead cells visualized by Phloxine B in the wild type (WT), *ccr4*Δ and *ccr4-tev* with or without Gar2-TEVp expression. Cells were cultured for 24 h in glucose-poor medium (*n* > 200 cells).

We first knocked out the *ago1*^+^ gene and examined H3K9me2 levels in rDNA. We found that H3K9me2 levels were reduced in *ago1*Δ irrespective of glucose concentration (Figure 5A). We also studied the effects of the deletion of *ccr4*^+^, which also contributes to the RNAi pathway (24), and found that the absence of *ccr4*^+^ resulted in the attenuation of H3K9me2 in rDNA (Figure 5A). These results support that the RNAi-dependent pathway is involved in the rDNA histone methylation. It should be noted that similar levels of reduction were observed upon double deletion of *ago1*^+^ and *ccr4*^+^ (Figure 5A), suggesting that Ago1 and Ccr4 function in the same pathway.

We further examined whether the RNAi-dependent pathway affects the recruitment of Nuc1, a component of RNA polymerase I in rDNA (Figure 5B). We observed an increase of Nuc1-binding to rDNA in *ccr4*Δ and *ago1*Δ as compared to the wild type under both glucose-rich and -poor conditions (Figure 5B). Accordingly, 18S rRNA abundance in both *ccr4*Δ and *ago1*Δ was markedly higher than that in the wild type under glucose-rich conditions (Supplementary Figure S5A). In addition, during glucose starvation, the 18S rRNA abundance was less reduced in *ccr4*Δ and *ago1*Δ than in the wild type (Supplementary Figure S5B). Therefore, it is suggested that the RNAi-dependent pathway is involved in heterochromatinization and gene silencing of rDNA. On the other hand, it should be noted that Nuc1 binding in rDNA was gradually reduced during glucose starvation in the wild type, *ccr4*Δ and *ago1*Δ (Figure 5B). These results suggest that RNAi-independent pathways might also contribute to the repression of RNA polymerase I (Nuc1)-dependent rRNA transcription (see Discussion).

It was previously shown that H3K9 methylation is also promoted by the MTREC (Mtl1-Red1 complex)-mediated RNAi-independent pathway in heterochromatin islands often found in meiotic-specific loci (25,27,82). Therefore, we investigated whether MTREC contributes to H3K9 methylation in rDNA. ChIP experiments revealed that Red1 was more enriched in rDNA than in other control loci (Supplementary Figure S5C). H3K9me2 in 18S rDNA region was markedly reduced in *red1*Δ especially at early stages of glucose starvation (Supplementary Figure S5D), but it eventually increased to the same extent as in the wild type. This delayed increase of H3K9me2 was not observed in the double deletion of *ccr4*^+^ and *red1*^+^ (Supplementary Figure S5D), suggesting that MTREC contributes to H3K9 methylation mainly in glucose-rich conditions. Taken together, these results indicate that the RNAi-dependent and RNAi-independent pathways redundantly contribute to H3K9 methylation in rDNA.

### A lack of starvation-induced H3K9 methylation leads to cell death

In this study, we uncovered the mechanisms responsible for H3K9 methylation in rDNA during glucose starvation. Therefore, we next pursued the biological significance of starvation-induced rDNA heterochromatinization. When cells are spotted onto plates containing Phloxine B, dead cells form red-colored colonies. The *ccr4*Δ mutant produced darker red colonies than the wild type under glucose-starved conditions (Figure 5C). We measured the percentage of dead cells by microscopy and found that the *ccr4*Δ mutant formed about three times more dead cells than the wild type (Figure 5D and Supplementary Figure S5E). Similar results were indicated by measuring cell viability on agar plates. Exposure of the *ccr4*Δ, *ago1*Δ and *clr4*Δ cells to a glucose-starved environment for 1 or 3 days resulted in markedly reduced viability compared to the wild type (Figure 5E and F). On the other hand, the *pob3*Δ and *atf1 o.p* strains, which exhibited reduced heterochromatin formation, did not show a marked loss of viability under glucose-poor conditions after one or three days of glucose depletion (Supplementary Figure S5F). However, cell growth of the *pob3*Δ and *atf1 o.p* strains was slower as compared to the wild type during glucose starvation (Supplementary Figure S5G). Altogether, disruption of starvation-induced H3K9 methylation affects cell fitness.

However, we cannot exclude the possibility that the reduction of cell viability is due to side effects of these deletions rather than specific defects caused by a lack of rDNA heterochromatinization. We therefore tested whether cell viability is restored if we introduce an additional mutation in RNA polymerase I (*nuc1-632*), since *nuc1-632* is known to reduce rDNA expression (83,84), and is expected to compensate for the loss of heterochromatinization in rDNA. We found that cell viability in *ccr4*Δ, *ago1*Δ, and *clr4*Δ was largely restored by the *nuc1-632* mutation (Figure 5G), which repressed the binding of Nuc1-FLAG to rDNA regions in *ccr4*Δ (Figure 5B).

In addition, we examined whether cell viability deteriorated if we degraded Ccr4 locally in rDNA using the TEV protease system. Ccr4-tev-HA was cleaved by TEV protease *in vitro* (Supplementary Figure S5H). As observed in Gcn5-tev-HA (see Figure 4C), any fragments of Ccr4-tev-HA by Gar2-TEV protease were not detectable *in vivo* (Figure 5H), suggesting that the amount of Ccr4 localized in the nucleolus was very limited when compared to the bulk of Ccr4-tev-HA. Notably, ChIP-qPCR revealed that the amount of Ccr4 in rDNA was indeed reduced when the TEV protease was co-expressed (Figure 5I), but not in other regions with potential Ccr4-dependent H3K9 methylation (24). The strain expressing Ccr4-tev-HA and Gar2-TEV protease generated many dead cells under glucose-poor conditions (Figure 5J), as observed in *ccr4*Δ. These results suggest that the loss of heterochromatinization in rDNA leads to cell death during glucose starvation. These results led us to conclude that the silencing of rDNA is essential for cellular adaptation and survival during glucose starvation.

Additionally, *atf1*-deleted cells, in which heterochromatin was excessively formed in rDNA irrespective of nutritional conditions, proliferated more slowly and more frequently showed red-colored colonies on plates containing Phloxine B than the wild type (Supplementary Figure S5I, J). We also found that *pob3Δ* and *atf1* overexpressed strains, in which rRNA transcriptions were substantially increased under glucose-rich conditions, grew more slowly than the wild type (Supplementary Figure S5I). Therefore, the proper amount of rDNA transcription is also crucial for optimal cell growth under glucose-rich conditions.

## DISCUSSION

Previous studies have demonstrated that cells quickly adapt to harsh environments by modulating their gene expression patterns in response to stress. The transcription pattern in fission yeast is largely altered in response to glucose starvation, a phenomenon that is driven in major part by the transcription factor Atf1, which binds upstream of stress-responsive genes to activate transcription by recruiting chromatin modifiers such as Gcn5 (37,38). On the other hand, while many ribosome-related genes are repressed in the early response to glucose starvation, the mechanisms underlying this gene repression had not yet been elucidated. In this study, we uncovered the molecular mechanisms directing rDNA gene repression in response to glucose starvation. Moreover, we found that this starvation-induced rDNA silencing is essential for cell survival during glucose starvation.

### Roles of Atf1 in the rDNA heterochromatinization

Our study unveiled the hidden importance of Atf1 dissociation in gene silencing. A previous study had found 72 genes (including *rps2602*^+^ encoding the 40S ribosomal protein) from which Atf1 dissociates in response to oxidative stress in fission yeast (65). We found that Atf1 is dissociated from 554 genes after glucose depletion (Figure 3A), 34 of which also show a decrease in transcript expression levels (Figure 3C). We therefore consider that the gene silencing mechanism based on Atf1 dissociation is in a subset of genes for stress-response and ribosome biogenesis.

Intriguingly, Atf1 was widely accumulated in rDNA and ribosome-related genes (*rpl102*^+^, *rlp7*^+^ and *gar2*^+^). It is well known that TGACGT is the 6-bp consensus sequence (*CRE* core sequence) where Atf1 binds (85,86). In fact, *CRE* core sequences are present in three loci within one repeat of rDNA. However, they are seemingly absent in the *rpl102*^+^, *rlp7*^+^ and *gar2*^+^ genes. Another study has proposed that Atf1 might bind to *CRE*-like sequences and induce recombination there (87). Therefore, Atf1 may recognize *CRE*-like sequences in those loci.

Alternatively, Atf1 may form a complex with protein(s) that contain a DNA-binding motif in those loci even in the absence of *CRE* core sites. Indeed, Atf1 interacts with the DNA-binding transcription factor Cbf11 (88). Alteration of such complex formation of Atf1 with DNA-binding factor(s) in response to stress may lead to dissociation of Atf1 from chromatin in rDNA.

Signal transduction pathways controlling the rDNA heterochromatinization would be another interesting aspect. Atf1 dissociated from rDNA even in the *spc1*Δ strain (Supplementary Figure S2B), indicating that other signal transduction pathways such as mTOR and AMPK may be important. It should be pointed out that yeast and mammalian TORC1 accumulate to rDNA (89,90), suggesting that it may contribute to rDNA gene regulation. Interestingly, TORC1 is inactivated by high-temperature stress (91). Since we observed heterochromatinization in rDNA under heat stress, TORC1 is expected to be involved in the regulation of rDNA heterochromatinization.

The data in this study suggest starvation-induced dissociation of Atf1 from rDNA causes the rDNA heterochromatinization. How does Atf1 control this process? Previous studies have suggested that Atf1 contributes to both acetylation and methylation of histone H3 by recruiting the acetyltransferase Gcn5 and the methyltransferase Clr4, respectively. Atf1 activates stress response genes through Gcn5 and silences mating-type loci through Clr4 (37,57,58,92,93). In this study, we found a novel mechanism by which Atf1 dissociation promotes local H3K9 methylation by recruiting FACT (Spt16–Pob3 complex) (Figure 2J), which in turn prevents the turnover/eviction of methylated histone H3K9 (14). H3K9 methylation was reduced in *pob3*Δ both under glucose-rich and -poor conditions (Supplementary Figure S2F), suggesting that FACT regularly contributes to the modulation of methylated H3K9 in rDNA, which reportedly exhibits rapid turnover in mammalian cells (94). Upon glucose starvation, Atf1 dissociation leads to recruitment of FACT to rDNA, which further promotes H3K9 methylation.

### Dissociation of Gcn5 from rDNA

Our results also suggest that dissociation of Gcn5 in response to glucose depletion promotes heterochromatinization and silencing of rDNA. Targeted degradation of Gcn5 in the nucleolus leads to local histone methylation of H3K9 in rDNA even in glucose-rich conditions (Figure 4E). We thus consider that the process of Gcn5 dissociation may be a key step in the control of heterochromatinization and silencing of rDNA. Since Atf1 is known to recruit Gcn5 HAT to stress-responsive genes, we considered whether Atf1 dissociation is a prerequisite for the dismantlement of Gcn5 from rDNA. However, Gcn5 was for the most part normally dissociated from rDNA even in *atf1*Δ (Figure 2I), indicating that Atf1 is dispensable for Gcn5 dissociation.

**Figure 6.**
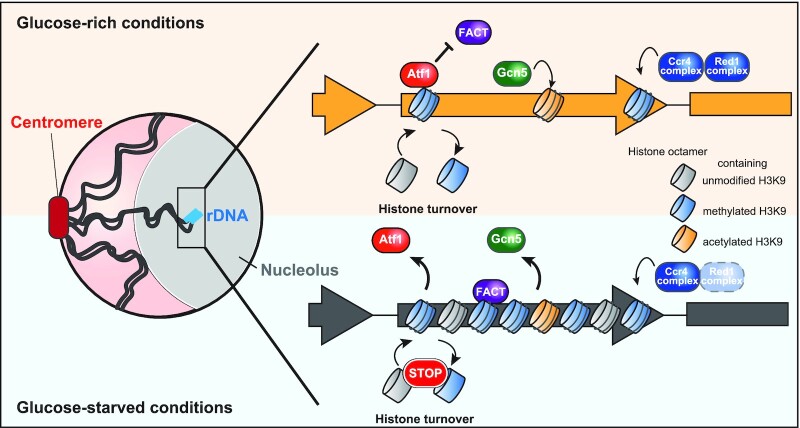
A model of heterochromatinization in rDNA during glucose starvation. (Upper) Glucose-rich conditions: accumulated Atf1 in rDNA inhibits the recruitment of FACT, resulting in acceleration of histone turnover, and thereby H3 density becomes lower, though RNAi-dependent and RNAi-independent pathways consistently mediate histone methylation. Enriched Gcn5 also locally inhibits H3K9 methylation. (Bottom) Glucose-starved conditions: dissociation of Atf1 leads to the recruitment of FACT to rDNA. FACT in turn prevents histone turnover and enhances H3 deposition. H3K9 methylation is induced promptly and mainly by RNAi-dependent pathway and is also supported by dissociation of Gcn5.

Another possibility is that the alteration of local histone modification can modulate the dissociation of Gcn5. Gcn5 carries a bromodomain which specifically binds acetylated histone H3, and hence can interact with the rDNA region in an Atf1-independent manner (95). The simplest model would be one in which H3K9ac levels control the dissociation of Gcn5. Indeed, HDACs Sir2 and Hst2 contribute to the silencing of rDNA (75). However, H3K9ac levels remained unchanged in H3K9-HDAC (Sir2 and Hst2) mutants. In addition, our data show that H3K9ac levels in rDNA were relatively unaffected by external glucose concentration (Supplementary Figure S4C). These results suggest that H3K9ac levels may have no connection with the dissociation of Gcn5 and subsequent H3K9 methylation. This idea is also consistent with previous observations in a deletion mutant of the budding yeast *RPD3* HDAC. The disruption of *rpd3* alleviates the silencing of rDNA regions, but more importantly does not affect the acetylation levels of H3K9-14 in rDNA (96). Gcn5 dissociation may be controlled by other histone modifications and H3K9 methylation of rDNA may be introduced via methylation of unmodified free H3 molecules (see Figure 6).

It should be noted that H3K9me2 in rDNA was almost completely lost in the deletion of *clr3* (Supplementary Figure S4D). Clr3 HDAC catalyzes the deacetylation of H3K14 leading to subsequent ubiquitination of H3K14 that promotes Clr4-mediated H3K9 methylation (17,74,78–80). Gcn5 is known to acetylate H3K14 (71,97), therefore acetylation of H3K14 may control the dissociation of Gcn5 from rDNA, but this idea needs to be further validated.

### Multiple mechanistic layers for starvation-induced rDNA silencing

A previous study had indicated that the methylation of H3K9 in rDNA regions requires the RNAi-dependent pathway (24). In fact, we observed a decrease in methylated H3K9 when *ago1*Δ or *ccr4*Δ cells were cultured both in glucose-rich and -poor media (Figure 5A), expecting that the RNAi-dependent mechanism constitutively supplies methylated histones irrespective of nutritional conditions. This assumption is supported by the fact that histone H3 density in rDNA is low under nutrient-rich conditions, whereas the level of H3K9 methylation is higher than in other regions (Figure 1E and F). The dynamic changes in H3K9 methylation in response to glucose depletion are mainly supported by the modulation of histone turnover by FACT as described above.

Why is heterochromatin weakly formed in rDNA even under nutritious conditions? One possible reason is that since the *ccr4* disruption causes an alteration in the number of rDNA repeats through mitotic recombination, heterochromatin formation may be required for rDNA integrity (24). Another possible idea is that heterochromatin formation prevents excessive rRNA transcription which causes delayed cell proliferation or toxic effects on cell viability. Indeed, *ccr4*Δ, which shows increased 18S rRNA abundance under glucose-rich conditions (Supplementary Figure S5A), exhibited an increase in dead cells in a glucose-rich medium (Figure 5D).

In addition to the RNAi-dependent pathway, RNAi-independent heterochromatinization is also involved through MTREC (Mtl1-Red1), which accumulates in rDNA and also contributes to H3K9 methylation (Supplementary Figure S5C and D). However, Sugiyama and his coworkers previously demonstrated by ChIP-chip experiments that H3K9 methylation in rDNA was not affected in *red1*Δ (24). The discrepancy could be explained by the difference in glucose concentration used, as our experiments employed a much higher glucose concentration. We confirmed that *red1*Δ cells exhibited the enhancement of methylated H3K9 in rDNA in a lower glucose concentration.

We showed that rDNA heterochromatinization prevents the accumulation of RNA polymerase I in rDNA. However, *ccr4*Δ showed less binding of Nuc1 in rDNA during glucose starvation, although *ccr4*Δ confers marked reduction of the rDNA heterochromatinization. Therefore, it is likely that other mechanisms are also involved in rDNA gene silencing. In budding yeast, inhibition of TOR (Target Of Rapamycin) by rapamycin or nutrient starvation leads to nucleolar condensation, which induces dissociation of RNA polymerase I from rDNA resulting in transcriptional silencing (98). Nutrient starvation is known to induce rDNA repositioning and nucleophagic degradation of nucleolar proteins, both of which cause transcriptional repression independently of rDNA heterochromatinization (99). Thus, multiple layers of mechanisms are involved in starvation-induced rDNA silencing.

### Biological significance of rDNA silencing during starvation

We revealed that disruption of heterochromatin formation in rDNA significantly reduces viability under glucose-limited conditions. Meanwhile, excessive heterochromatinization in rDNA can lower cellular viability during the G0 phase (42,100). Consistent with this, we also found that *atf1*Δ cells, in which excessive heterochromatin was formed in rDNA, exhibited loss of viability and cell fitness (Supplementary Figure S5I and J). These observations suggest that the strict control of rDNA transcription in response to environmental changes is vital for cellular survival. This is because ribosomes for protein synthesis account for about half of the entire gene expression activity in cells, and most of the nutrient and energy resources are utilized for ribosome synthesis.

In higher eukaryotes, misregulation of rRNA expression leads to a pathologic state called ‘ribosomopathy’. A number of human diseases caused by loss of ribosome function have been reported (101). In addition, zebrafish with insertion mutations in ribosome-associated genes are more likely to develop malignant peripheral nerve sheath tumors and other cancers (102). Our study newly sheds light on the importance of epigenetic regulators involved in ribosomal gene regulation. Further studies on the mechanisms of environment-dependent epigenetic gene silencing of ribosomes in higher eukaryotes are expected to open the way for novel molecular therapies for human ribosomopathy.

## DATA AVAILABILITY

The ChIP-seq data is available at the NCBI Sequence Read Archive database under accession number; SRR15056543, SRR15056539, SRR15056538 and SRR15056536.

## Supplementary Material

gkac175_Supplemental_File

## Notes

Present address: Naomichi Takemata, Department of Synthetic Chemistry and Biological Chemistry, Graduate School of Engineering, Kyoto University, Katsura, Nishikyo-ku, Kyoto 615-8510, Japan.
