# Supplementary material for: Facultative heterochromatin formation in rDNA is essential for cell survival during nutritional starvation
Source: Nucleic Acids Res. 2022 Mar 28;50(7):3727–44. doi: 10.1093/nar/gkac175 (PMC9023297; doi:10.1093/nar/gkac175)
Supplement: gkac175_Supplemental_File [file gkac175_supplemental_file.pdf]

Supplemental information

Facultative heterochromatin formation in rDNA is essential for cell survival during nutritional starvation

Hayato Hirai, Naomichi Takemata, Miki Tamura and Kunihiro Ohta

Supplementary Figure S1-S5

Supplementary Table S1-S5

Supplementary Figure S1

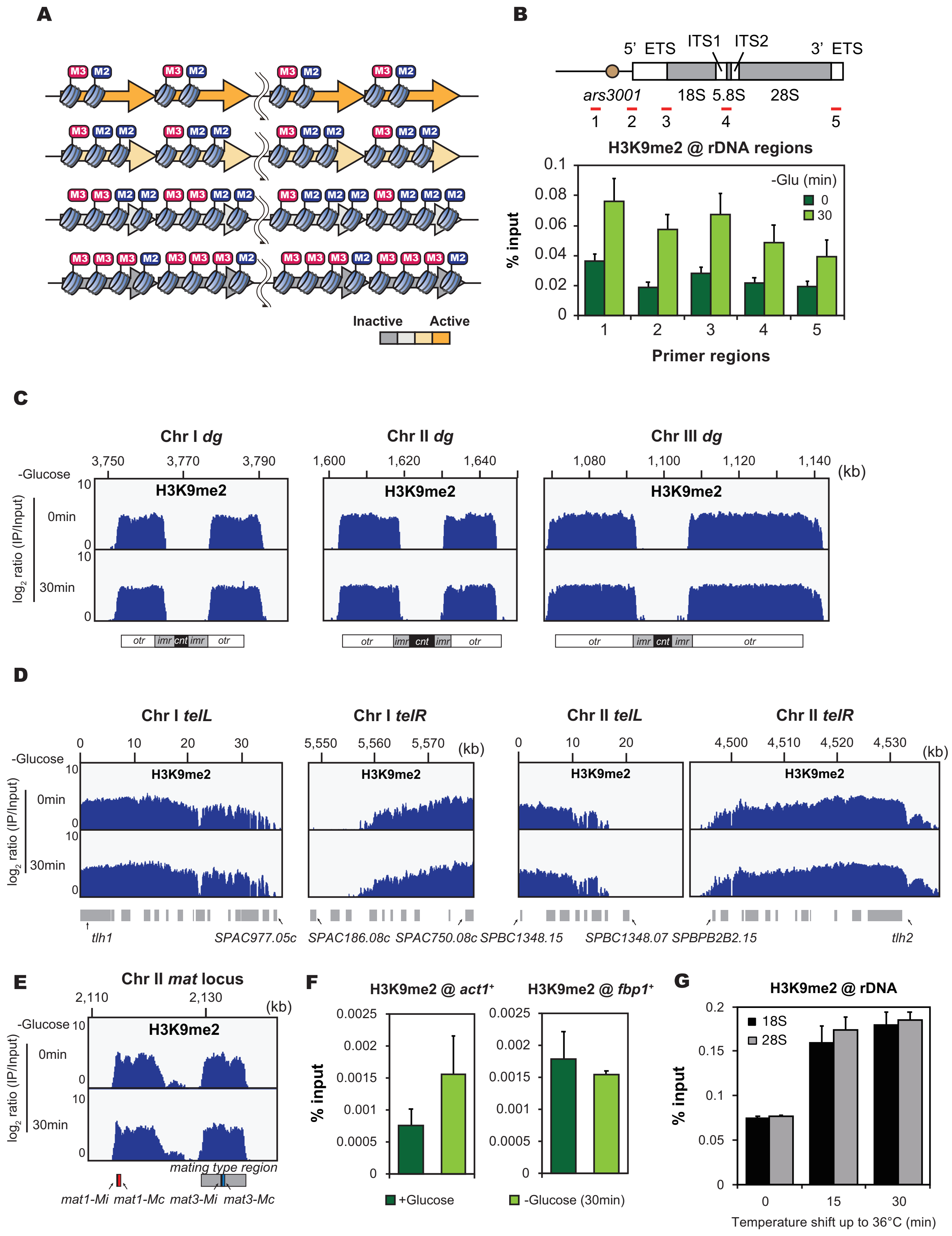

Supplementary Figure S1. Related to Figure 1.

(**A**) A schematic diagram showing the induction of H3K9 methylation in rDNA by glucose starvation. Methylated H3 may accumulate in specific regions in rDNA repeats even under nutritious conditions. In response to glucose starvation, histone H3 is incorporated into rDNA repeats and simultaneously dimethylated. Then, dimethylation is converted to trimethylation to enhance the heterochromatin state. (**B**) (top) A schematic diagram of the rDNA region with primer sets 1-5 (red lines) for ChIP-qPCR. (bottom) Analysis of the H3K9me2 levels in each region. -Glu indicates 0 or 30 min after glucose starvation (mean + SEM: n = 3). (**C**-**E**) ChIP-seq data showing H3K9me2 enrichment at peri-centromeres on chromosome I, II and III (**C**), and at sub-telomeres on chromosome I and II (**D**), and at mating type locus on chromosome II (**E**) during glucose-rich (0min, upper) and glucose-poor (30min, bottom) conditions. The y-axis exhibits log2 ratio of IP/input (0-10). Under panels, cnt: central core, imr: inner repeat region, otr: outer repeat region. Grey boxes indicate genes. (**F**) ChIP-qPCR showing H3K9me2 levels in euchromatin regions (the *act1*$^{+}$ coding sequence and the upstream of *fbp1*$^{+}$). Glucose starvation was induced for 30 min (mean + SEM: n = 3). (**G**) ChIP-qPCR showing the levels of H3K9me2 in 18S and 28S rDNA (mean + SEM: n = 3). Cells were harvested 0, 15, and 30 min after temperature shift up to 36°C.

Supplementary Figure S2

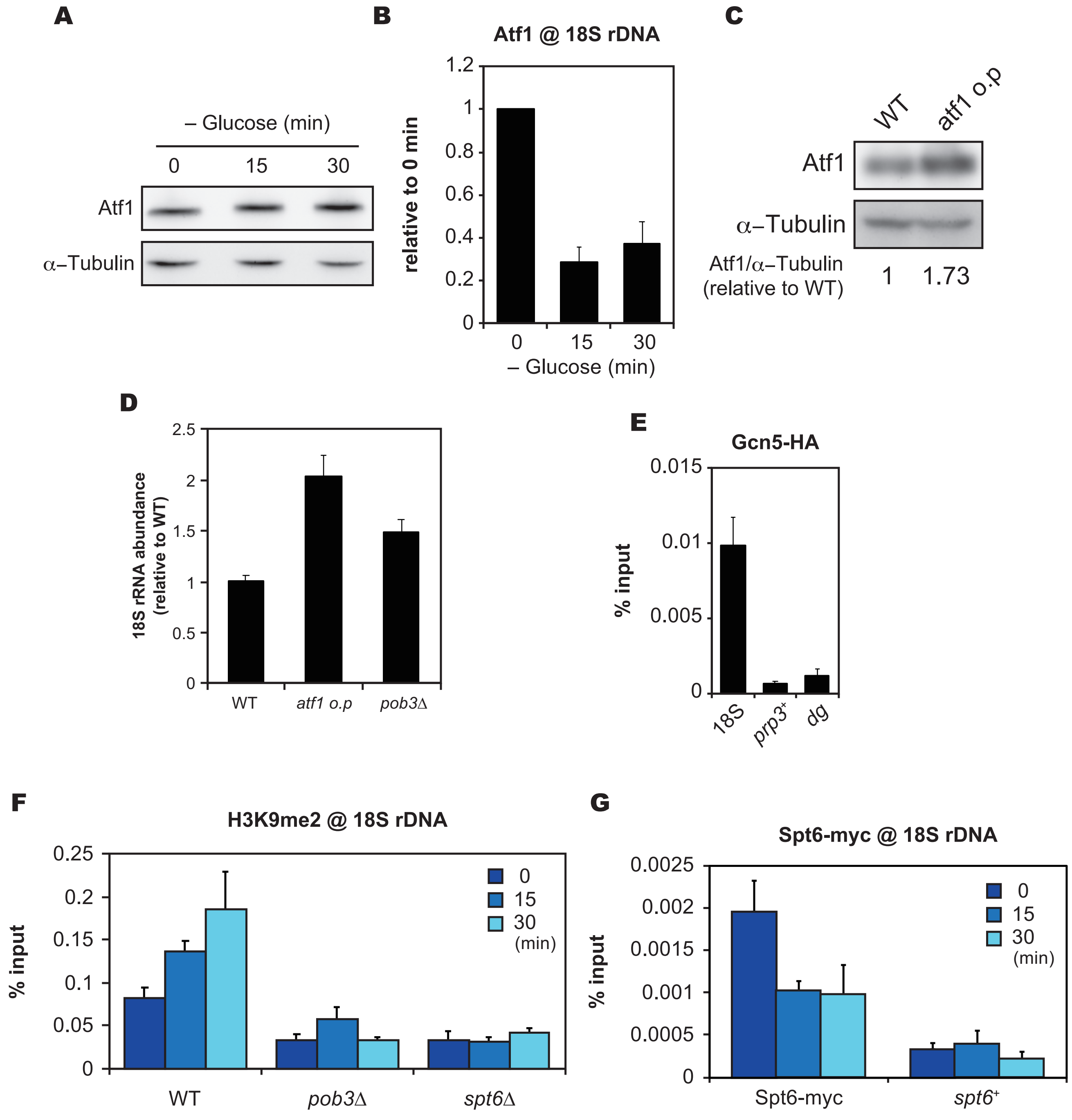

Supplementary Figure S2. Related to Figure 2.

(**A**) The amount of bulk Atf1 in cell extracts is unchanged by glucose starvation. Immunoblotting was performed with anti-Atf1 and anti-α-Tubulin antibodies. (**B**) Atf1 binding to 18S rDNA in the *spc1Δ* strain. Atf1 dissociated from rDNA after glucose starvation like in the wild type. Values relative to that at 0 min after glucose starvation are indicated (mean + SEM: n = 3). (**C**) Immunoblotting examining the amount of Atf1 and α-Tubulin (top). Signal intensity ratio of Atf1/α-Tubulin in the wild type (WT) and *atf1 o.p* strain (bottom). Signal intensity of bands was measured by ImageJ. (**D**) 18S rRNA abundance relative to the wild type expression level (WT) under glucose-rich conditions in *atf1 o.p* and *pob3Δ* strains (mean+ SD: n = 3). (**E**) ChIP-qPCR showing Gcn5-HA enrichment in 18S rDNA, *prp3*$^{+}$ and *dg* (peri-centromeres) regions (mean + SEM: n = 3). The data for 18S rDNA is the same as in Figure 2I. (**F**) H3K9me2 levels in 18S rDNA in the wild type, *pob3Δ* and *spt6Δ* (mean + SEM: n = 3). The data for the wild type is identical to that in Figure 2C. (**G**) ChIP-qPCR quantifying Spt6-myc occupancy in 18S rDNA during glucose starvation. Control experiments were conducted using an untagged strain (*spt6*$^{+}$) (mean + SEM: n = 3).

Supplementary Figure S3

A

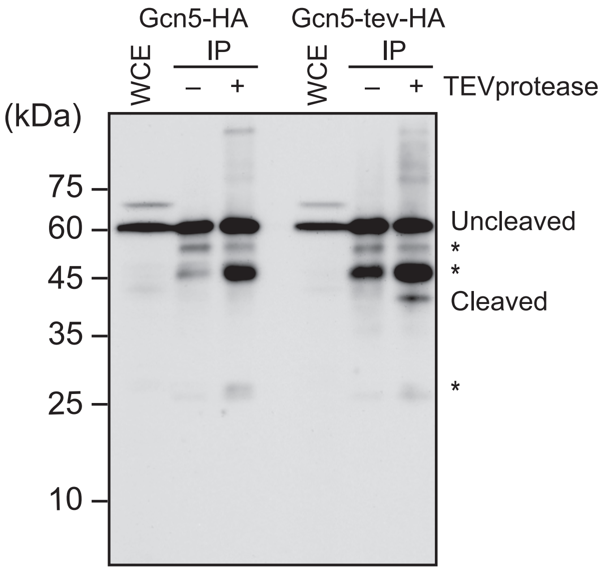

C

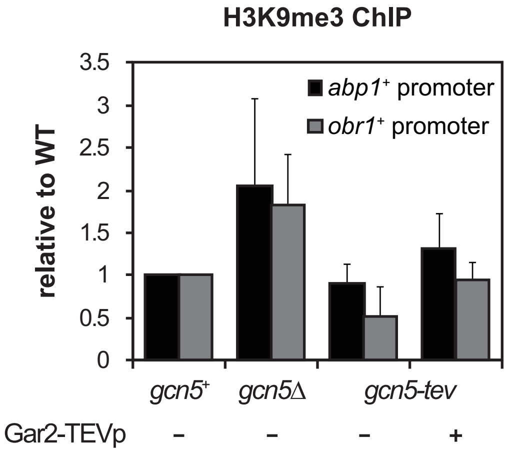

B

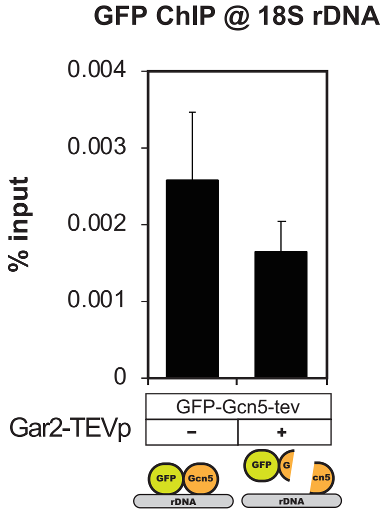

Supplementary Figure S3. Related to Figure 4.

(**A**) Immunoblotting showing the cleaved Gcn5-tev-HA. Immunoprecipitated Gcn5-HA and Gcn5-tev-HA were incubated with (+) or without (–) TEV protease at 30˚C overnight. The full-length Gcn5-tev-HA (Uncleaved) and the cleaved products (Cleaved) were detected. Asterisks indicate non-specific bands. (**B**) ChIP-qPCR showing the amount of GFP-Gcn5-tev in 18S rDNA when Gar2-TEV protease is co-expressed (+) or not (-) (mean + SEM: n = 12). (**C**) ChIP-qPCR showing H3K9me3 levels at the *abp1*$^{+}$ and *obr1*$^{+}$ promoters in the wild type, *gcn5*Δ, *gcn5-tev-HA* and *gcn5-tev-HA gar2-TEVp* strains (mean + SEM: n = 3). Expression of *abp1*$^{+}$ and *obr1*$^{+}$ genes is known to be activated by Gcn5.

Supplementary Figure S4

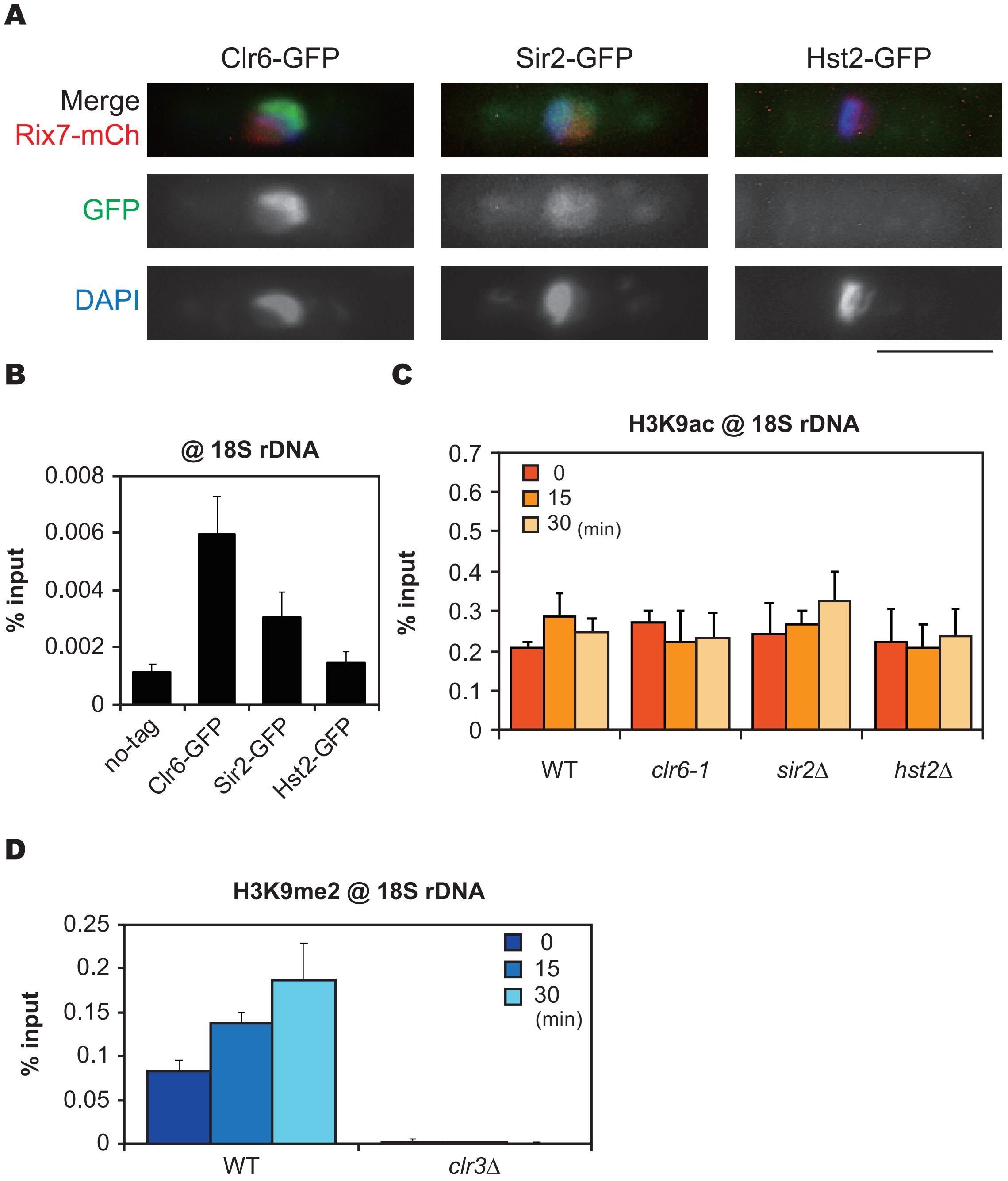

Supplementary Figure S4. Roles of HDACs in the rDNA heterochromatinization.

(**A**) Localization of Clr6-GFP, Sir2-GFP and Hst2-GFP (green). Nucleoli and DNA were visualized with Rix7-mCherry (red) and DAPI (blue), respectively. Scale bar = 5 μm. (**B**) ChIP-qPCR data showing enrichment of Clr6-GFP (n = 3), Sir2-GFP (n = 5) and Hst2-GFP (n = 3) in 18S rDNA. No-tag indicates the wild type without GFP expression (WT, n = 4), (mean + SEM). (**C**) H3K9ac levels in 18S rDNA in the wild type (WT), *clr6-1*, *sir2*Δ and *hst2*Δ mutants during glucose starvation (mean + SEM: n = 3). (**D**) H3K9me2 levels in 18S rDNA were drastically reduced in the *clr3*Δ mutant (mean + SEM: n = 3). The data for the wild type (WT) is identical to that in Figure 2C.

Supplementary Figure S5

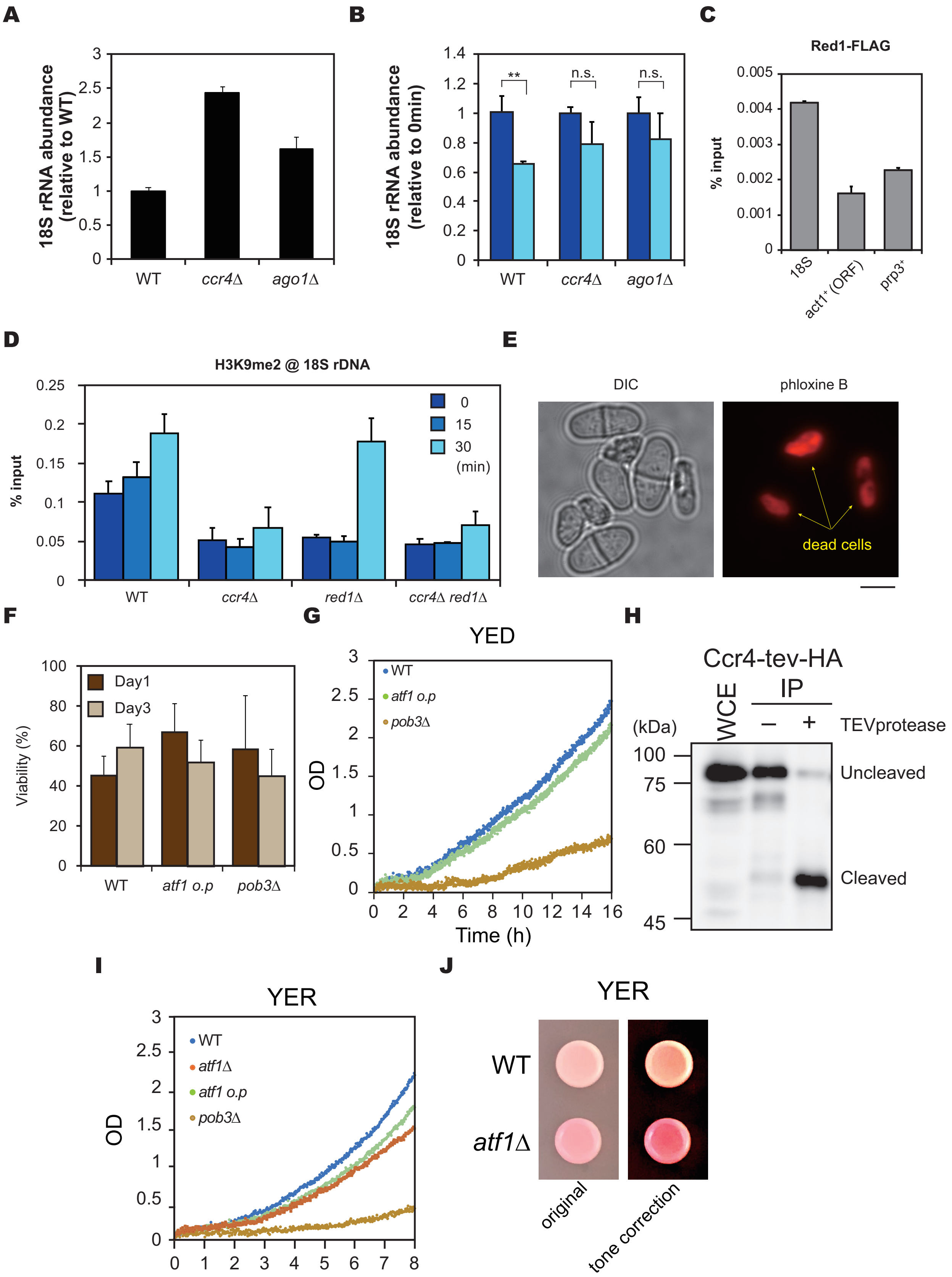

Supplementary Figure S5. Related to Figure 5.

(**A**) 18S rRNA abundance relative to the wild type expression level (WT) under glucose-rich conditions in *ccr4*Δ and *ago1*Δ mutants (mean + SD: n = 3). The wild type control data is the same as in Supplementary Figure S2D. (**B**) 18S rRNA abundance in the wild type (WT), *ccr4*Δ and *ago1*Δ mutants (mean + SD: n = 3) relative to those at 0 min. Student's *t*-test (two-tailed) was applied and statistical significance was indicated (**p<0.01, n.s., not significant). The wild type control data is the same as in Figure 2G. (**C**) ChIP-qPCR to quantify Red1-FLAG occupancy in 18S rDNA, $prp3^+$ and the coding region of $act1^+$ (mean + SEM: n = 5). (**D**) ChIP-qPCR results showing H3K9 methylation levels in 18S rDNA in the wild type (WT, n = 4), *ccr4*Δ (n = 3), *red1*Δ (n = 3) and *ccr4*Δ *red1*Δ (n = 3) mutants (mean + SEM). Time indicates minutes from glucose depletion. The data for the wild type is identical to that in Figure 5A. (**E**) Microscopic observation showing dead cells in the glucose-starved medium stained with Phloxine B. Dead cells (yellow arrows) are detected at 530 nm wavelength. (**F**) Viability data during glucose starvation in the wild type (WT, n = 3), *atf1 o.p* (n = 4) and *pob3*Δ (n = 4) strains (mean + SD). The data for the wild type is identical to that in Figure 5F. (**G**) Monitoring of cell density (OD) per minute in the wild type (WT), *atf1 o.p*, and *pob3*Δ strains. Times after glucose depletion are indicated. (**H**) Immunoblotting showing cleaved Ccr4-tev-HA. Immunoprecipitated Ccr4-tev-HA was incubated with (+) or without (–) TEV protease at 30°C overnight. The band of full-length Ccr4-tev-HA (Uncleaved) disappeared and the cleaved product (Cleaved) was detected when TEV protease was added. (**I**) Monitoring of cell density (OD) per minute in the wild type (WT), *atf1*Δ, *atf1* overexpressed (*atf1 o.p*) and *pob3*Δ cells. Cells were grown in a glucose-rich medium (YER) and culturing time is indicated. (**J**) Spot assay. 10^6 cells were spotted on the glucose-rich YER agar plate containing phloxine B. Cells lacking $atf1^+$ exhibited a red-colored spot more frequently than the wild type (WT) cells. Original and tone-corrected images are shown.

Supplementary Table S1. List of strains used in this study

Strain name	Genotype	Figures
L975	h^+	1A-F, K, 2B, D, F, G, 3E, 4A, C, 5A, C-F, H, J, S1B, F, G, S2A, C, D, G, S3C, S4B-D, S5A, B, D, F, G, I, J
HH187	h^+ *pob3-FLAG-kan*	1G, S1C-E
HH42	*mat1M-Smt0 GFP-swi6-kan*	1H, I
HH161	h^+ *nuc1-FLAG-kan*	1J, 5B
HH21	h^+ *gcn5-3HA-kan*	2C, E, H, I, S2E, F
HH51	h^+ *gcn5-3HA-kan atf1::ura4+ ura4-D18*	2C, I
HH104	h^+ *Z::adh21-atf1-nat*	2D, E, G, S2C, D, S5F, G, I
HH18	h^+ *atf1::ura4+ ura4-D18*	2F, 3E, S5I, J
HH153	h^+ *pob3::kan*	2G, S2D, F, S5F, G, S5I
HH84	h^+ *pob3-GFP-kan*	2J
HH92	h^+ *pob3-GFP-kan atf1::hph*	2J
HH276	h^+ *pob3-GFP-kan Z::adh21-atf1-nat*	2J
HH26	h^+ *gcn5::ura4+ ura4-D18*	4A, S3C
HH95	h^+ *gcn5-tev(148)-3HA-hph*	4C-E, S3A, S3C
HH99	h^+ *gcn5-3HA-hph*	4C, S3A
HH100	h^- *gar2-TEVprotease-kan gcn5-tev(148)-3HA-hph*	4C-E, S3C
HH106	h^+ *gar2-TEVprotease-kan gcn5-3HA-hph*	4C-E
HH73	h^+ *ago1::hph*	5A, F, S5A, B
HH80	h^+ *ccr4::kan*	5A, S5A, B
HH93	h^+ *ccr4::kan ago1::hph*	5A
HH163	h^+ *nuc1-FLAG-kan ccr4::hph*	5B
HH199	h^+ *nuc1-632-FLAG-kan ccr4::hph*	5B
HH224	h^+ *nuc1-FLAG-kan ago1::hph*	5B
NT322	*mat1M-Smt0 ccr4::hph*	5C-F, J, S5D, E
NT287	h^+ *clr4::kan*	5F
HH167	h^+ *nuc1-632 ago1::hph*	5G
HH172	h^+ *nuc1-632*	5G
HH175	h^+ *nuc1-632 ccr4::hph*	5G
HH181	h^+ *nuc1-632 clr4::kan*	5G
HH272	h^+ *ccr4-tev(300)-3HA-hph*	5H-J, S5H
HH285	h^+ *gar2-TEVprotease-kan ccr4-tev(300)-3HA-hph*	5H-J
HH81	h^- *spc1::ura4+ ura4-D18*	S2B
HH137	h^+ *spt6::ura4+ ura4-D18*	S2F
HH140	h^+ *spt6-13myc-hph*	S2G

Strain name	Genotype	Figures
HH165	h^+ *GFP-gcn5-tev(148)-3HA-hph*	S3B
HH173	h^+ *gar2-TEVprotease-kan GFP-gcn5-tev(148)-3HA-hph*	S3B
HH1090	h^- *clr6-GFP-kan leu1-32 ura4-D18 ade6-M216*	S4A, B
HH1140	h^- sir2-*GFP-kan leu1-32 ura4-D18 ade6-M216*	S4A, B
HH1142	h^- hst2-*GFP-kan leu1-32 ura4-D18 ade6-M216*	S4A, B
HH52	h^- *clr6-1*	S4C
HH57	h^- *hst2::ura4+ ura4-D18*	S4C
HH59	h^- sir*2::ura4+ ura4-D18*	S4C
HH260	h^+ *clr3::kan*	S4C
NT347	*mat1M-Smt0 red1-FLAG-kan 12myc-clr4*	S5C
HH127	h^+ *red1::kan*	S5D
HH129	*mat1M-Smt0 red1::kan ccr4::hph*	S5D

HH1090, HH1140, and HH1142 were distributed from M. Sato. FY21276 and FY21289 strains were provided from NBRP (originated from M. Yanagida), from which HH167, HH172, HH175, HH181, and 199 were created. HH52 was generated from HH1444 distributed by M. Sato.

Supplementary Table S2. List of primers used in this study

Number	Name	Sequence	Figures
NT2	fbp1-UAS1_S_-1223	CTCCTCCTACTAACTACTCCATCC	1E, F, S1F
NT4	fbp1-UAS1_A_-1142	TGATGAGTACTAATGCTTTGTGACG	1E, F, S1F
NT23	act1-ORF_F	CTCTGGTGATGGTGTTACCCACACTG	1E, F, S1F, S5C
NT24	act1-ORF_R	AAGTCACGACCGGCGAGATCAAG	1E, F, S1F, S5C
NT83	prp3_F	GCACAGTCGTTGTACAAATTCGTATTCCC	S2E, S5C
NT84	prp3_R	ACGATTCTAAACGCCTCTTGTTACGATCC	S2E, S5C
NT123	cen-dg_F	AATTGTGGTGGTGTGGTAATAC	1H, I, 5I, S2E
NT124	cen-dg_R	GGGTTCATCGTTTCCATTCAG	1H, I, 5I, S2E
NT125	mei4_F	CTTTCTTTACAAAAACATCGCGAAC	5I
NT126	mei4_R	TGTTAAATCAGGGACATAAAGAAGTGAC	5I
NT169	act1_RT-_F	TACCCCATTGAGCACGGTAT	1K, 2F, G, 3E, 5B, S2D, S5A
NT170	act1_RT_R	CTTCTCACGGTTGGATTTGG	1K, 2F, G, 3E, 5B, S2D, S5A
NT278	rDNA_N1_F	AGAAAAAGTCGAGCGAGTCG	S1B
NT279	rDNA_N1_R	TCTTCAAAAGTGCATTACCCTTAC	S1B
NT282	rDNA_N4_F	CACCGAAATGGACGAAATTCAC	S1B
NT283	rDNA_N4_R	CCTTGGACCTATTTTTCCTCGA	S1B
NT284	rDNA_18S-1_F	CGATAGTTACCTGGTTGATCCTG	S1B
NT285	rDNA_18S-1_R	TGAGCCATTCGCAGTTTCAC	S1B
NT286	rDNA_18S-2_F	TCGGCACCTTACGAGAAATC	1A-C, E, F, H-K, 2B-J, 4A, D, E, 5A, B, I, S1G, S2B, D-G, S3B, S4B-D, S5A-D
NT287	rDNA_18S-2_R	TATGTCTGGACCTGGTGAGT	1A-C, E, F, H-K, 2B-J, 4A, D, E, 5A, B, I, S1G, S2B, D-G, S3B, S4B-D, S5A-D
NT288	rDNA_5.8S_F	CAACGGATCTCTTGGCTCTC	1J, S1B
NT289	rDNA_5.8S_R	CGATGATTCACGGAATTCTGCA	1J, S1B
NT290	rDNA_28S-1_F	GAGACCGATAGCGAACAAGTAG	1A-C, J, 2H, S1G
NT291	rDNA_28S-1_R	TGACCTACCACAGGTTCAGAG	1A-C, J, 2H, S1G
NT307	rDNA_N3_F	TGAAGCGGGTGTAAAGATGAG	S1B
NT308	rDNA_N3_R	GAATGACTGTTTTTGCATTCCAG	S1B
NT330	mcp5_F	ATGAAACCGACAGAGATGATGAG	5I
NT331	mcp5_R	CAGAGAAAGTGGGTACAAGCAAG	5I
	abp1_Prom_Fw	CATTCTACCTGCACTTCATTTCG	S3C

Number	Name	Sequence	Figures
	abp1_Prom_Rv	ACAACAATGGAATGCTGGCC	S3C
	obr1_Prom_Fw	CAGTGCTGACCAATTCCTTGC	S3C
	obr1_Prom_Rv	TTTATCGATGTAGCTGCGCC	S3C
	rpl102-Fw	AATGTCCCCCGTCCTAACAT	3E
	rpl102-Rv	CAACAAACGAGGGATTTGCT	3E
	rlp7-Fw	CTACCGCCAACGAGAAAGAG	3E
	rpl7-Rv	AAGGCGTAATAAGCGCAGAA	3E
	gar2-Fw	CCGAATCCAGCTCTGAAGAC	3E
	gar2-Rv	CGTGCACCAACAATTGTACC	3E

The NT2 and NT4 paired primers are the same sequence as those used in (1).

The NT83 and NT84 paired primers are the same sequence as those used in (2).

The NT123-NT124 and NT125-126 paired primers are the same sequence as those used in (3).

The NT169 and NT170 paired primers are the same sequence as those used in (4).

Supplementary Table S3. Genes where Atf1 binding was reduced

No.	Systematic ID	No.	Systematic ID	No.	Systematic ID	No.	Systematic ID
1	SPAC1006.09	26	SPAC13G6.02c	51	SPAC19B12.04	76	SPAC23H3.13c
2	SPAC1039.02	27	SPAC13G6.06c	52	SPAC19B12.05c	77	SPAC23H4.06
3	SPAC1071.06	28	SPAC13G6.07c	53	SPAC19G12.05	78	SPAC23H4.07c
4	SPAC1071.07c	29	SPAC140.02	54	SPAC1B3.05	79	SPAC24C9.11
5	SPAC1071.09c	30	SPAC144.11	55	SPAC1F12.02c	80	SPAC24C9.12c
6	SPAC1071.10c	31	SPAC144.12	56	SPAC1F7.13c	81	SPAC24H6.07
7	SPAC10F6.01c	32	SPAC15E1.03	57	SPAC1F8.06	82	SPAC25B8.13c
8	SPAC10F6.02c	33	SPAC16.04	58	SPAC1F8.07c	83	SPAC25G10.06
9	SPAC110.01	34	SPAC16.05c	59	SPAC1F8.08	84	SPAC25G10.07c
10	SPAC110.03	35	SPAC1687.05	60	SPAC222.09	85	SPAC26A3.04
11	SPAC110.04c	36	SPAC1687.06c	61	SPAC222.11	86	SPAC26A3.07c
12	SPAC11E3.12	37	SPAC1705.03c	62	SPAC227.16c	87	SPAC26A3.17c
13	SPAC11E3.13c	38	SPAC17A2.11	63	SPAC22A12.14c	88	SPAC26F1.06
14	SPAC11E3.15	39	SPAC17A2.12	64	SPAC22A12.15c	89	SPAC26F1.14c
15	SPAC11G7.04	40	SPAC17A5.03	65	SPAC22G7.06c	90	SPAC29A4.02c
16	SPAC11G7.05c	41	SPAC17A5.04c	66	SPAC22H12.03	91	SPAC29B12.08
17	SPAC1250.05	42	SPAC17D4.01	67	SPAC22H12.04c	92	SPAC2C4.16c
18	SPAC12G12.04	43	SPAC17D4.02	68	SPAC23A1.07	93	SPAC2E1P3.04
19	SPAC12G12.05c	44	SPAC1805.10	69	SPAC23A1.08c	94	SPAC2E1P3.05c
20	SPAC1399.06	45	SPAC1805.11c	70	SPAC23A1.10	95	SPAC2F3.09
21	SPAC13C5.05c	46	SPAC1805.13	71	SPAC23A1.11	96	SPAC2F3.16
22	SPAC13C5.07	47	SPAC1805.14	72	SPAC23A1.12c	97	SPAC30D11.11
23	SPAC13D6.01	48	SPAC1834.13	73	SPAC23D3.12	98	SPAC31G5.03
24	SPAC13D6.02c	49	SPAC18G6.14c	74	SPAC23H3.08c	99	SPAC31G5.11
25	SPAC13G6.01c	50	SPAC19B12.02c	75	SPAC23H3.09c	100	SPAC31G5.17c

No.	Systematic ID	No.	Systematic ID	No.	Systematic ID	No.	Systematic ID
101	SPAC328.10c	126	SPAC4H3.10c	151	SPAC890.08	176	SPAPB17E12.13
102	SPAC3A12.10	127	SPAC513.01c	152	SPAC8C9.08	177	SPAPB17E12.14c
103	SPAC3A12.11c	128	SPAC521.05	153	SPAC8C9.09c	178	SPAPB18E9.05c
104	SPAC3F10.17	129	SPAC57A10.11c	154	SPAC8E11.10	179	SPAPB2B4.01c
105	SPAC3F10.18c	130	SPAC57A10.12c	155	SPAC8F11.10c	180	SPAPB8E5.05
106	SPAC3G6.05	131	SPAC589.09	156	SPAC9.09	181	SPAPB8E5.06c
107	SPAC3G6.11	132	SPAC589.10c	157	SPAC9.10	182	SPAPJ698.02c
108	SPAC3G9.03	133	SPAC5D6.01	158	SPAC926.03	183	SPBC106.18
109	SPAC3H1.07	134	SPAC5D6.02c	159	SPAC926.04c	184	SPBC1105.01
110	SPAC3H1.08c	135	SPAC644.15	160	SPAC959.05c	185	SPBC1105.02c
111	SPAC3H5.04	136	SPAC644.17c	161	SPAC959.07	186	SPBC1105.05
112	SPAC3H5.05c	137	SPAC664.03	162	SPAC959.08	187	SPBC11C11.08
113	SPAC3H5.07	138	SPAC664.05	163	SPAC959.09c	188	SPBC11C11.09c
114	SPAC3H5.10	139	SPAC664.06	164	SPAC977.16c	189	SPBC1271.09
115	SPAC3H5.11	140	SPAC664.07c	165	SPAC977.17	190	SPBC1289.03c
116	SPAC3H5.12c	141	SPAC694.04c	166	SPAC9E9.13	191	SPBC12C2.06
117	SPAC4D7.08c	142	SPAC694.05c	167	SPAC9G1.02	192	SPBC12C2.07c
118	SPAC4D7.09	143	SPAC6B12.15	168	SPAC9G1.03c	193	SPBC1347.10
119	SPAC4D7.10c	144	SPAC6F6.07c	169	SPAP8A3.02c	194	SPBC1347.13c
120	SPAC4F10.18	145	SPAC6G10.11c	170	SPAP8A3.06	195	SPBC13A2.03
121	SPAC4F10.22	146	SPAC6G9.08	171	SPAP8A3.07c	196	SPBC13A2.04c
122	SPAC4G9.07	147	SPAC6G9.09c	172	SPAPB15E9.01c	197	SPBC14C8.01c
123	SPAC4G9.08c	148	SPAC7D4.06c	173	SPAPB15E9.02c	198	SPBC14F5.05c
124	SPAC4G9.15	149	SPAC806.11	174	SPAPB15E9.06	199	SPBC15D4.05
125	SPAC4G9.16c	150	SPAC890.05	175	SPAPB17E12.05	200	SPBC1652.01

No.	Systematic ID	No.	Systematic ID	No.	Systematic ID	No.	Systematic ID
201	SPBC1683.09c	226	SPBC1861.01c	251	SPBC21C3.13	276	SPBC359.02
202	SPBC1683.10c	227	SPBC1861.02	252	SPBC21C3.14c	277	SPBC359.03c
203	SPBC1685.01	228	SPBC18E5.04	253	SPBC23G7.12c	278	SPBC365.03c
204	SPBC1685.02c	229	SPBC18E5.05c	254	SPBC23G7.14	279	SPBC3B8.02
205	SPBC1685.09	230	SPBC18E5.06	255	SPBC25H2.05	280	SPBC3B9.12
206	SPBC1685.10	231	SPBC18H10.11c	256	SPBC25H2.06c	281	SPBC3B9.13c
207	SPBC1685.11	232	SPBC18H10.12c	257	SPBC25H2.07	282	SPBC3D6.02
208	SPBC16C6.11	233	SPBC18H10.13	258	SPBC25H2.08c	283	SPBC3D6.04c
209	SPBC16C6.12c	234	SPBC18H10.14	259	SPBC26H8.06	284	SPBC3D6.15
210	SPBC16E9.02c	235	SPBC18H10.15	260	SPBC26H8.16	285	SPBC3H7.06c
211	SPBC16E9.05	236	SPBC1921.04c	261	SPBC27.08c	286	SPBC428.02c
212	SPBC16E9.06c	237	SPBC1921.05	262	SPBC29A3.04	287	SPBC428.11
213	SPBC16G5.13	238	SPBC19C2.07	263	SPBC29A3.05	288	SPBC4F6.04
214	SPBC16G5.14c	239	SPBC19F8.08	264	SPBC29A3.13	289	SPBC4F6.05c
215	SPBC1709.05	240	SPBC19G7.02	265	SPBC29B5.03c	290	SPBC4F6.15c
216	SPBC1709.06	241	SPBC19G7.03c	266	SPBC2F12.04	291	SPBC4F6.16c
217	SPBC1711.06	242	SPBC19G7.04	267	SPBC2F12.05c	292	SPBC56F2.02
218	SPBC1711.14	243	SPBC1D7.05	268	SPBC2F12.14c	293	SPBC56F2.12
219	SPBC1711.15c	244	SPBC211.02c	269	SPBC30B4.04c	294	SPBC577.02
220	SPBC1734.12c	245	SPBC216.02	270	SPBC30D10.18c	295	SPBC577.03c
221	SPBC17G9.07	246	SPBC21B10.10	271	SPBC317.01	296	SPBC685.06
222	SPBC17G9.08c	247	SPBC21B10.11	272	SPBC32H8.11	297	SPBC685.07c
223	SPBC17G9.10	248	SPBC21C3.08c	273	SPBC336.08	298	SPBC713.08
224	SPBC17G9.11c	249	SPBC21C3.09c	274	SPBC336.09c	299	SPBC776.02c
225	SPBC1815.01	250	SPBC21C3.10c	275	SPBC336.10c	300	SPBC776.11

No.	Systematic ID	No.	Systematic ID	No.	Systematic ID	No.	Systematic ID
301	SPBC776.12c	326	SPBPB2B2.05	351	SPCC1840.13	376	SPCC622.18
302	SPBC800.04c	327	SPCC1183.07	352	SPCC191.08	377	SPCC622.19
303	SPBC83.01	328	SPCC1183.08c	353	SPCC24B10.09	378	SPCC663.04
304	SPBC83.02c	329	SPCC11E10.01	354	SPCC24B10.10c	379	SPCC663.18
305	SPBC839.05c	330	SPCC1259.01c	355	SPCC24B10.21	380	SPCC74.05
306	SPBC839.12	331	SPCC1281.08	356	SPCC285.14	381	SPCC794.08
307	SPBC839.13c	332	SPCC1322.11	357	SPCC285.15c	382	SPCC794.09c
308	SPBC839.20	333	SPCC1322.12c	358	SPCC31H12.04c	383	SPCC825.05c
309	SPBC8D2.05c	334	SPCC1393.03	359	SPCC330.13	384	SPCC970.05
310	SPBC8D2.17	335	SPCC13B11.01	360	SPCC364.03	385	SPCP1E11.08
311	SPBC8E4.01c	336	SPCC1450.04	361	SPCC364.04c	386	SPCP31B10.07
312	SPBP23A10.10	337	SPCC1450.05c	362	SPCC364.07	387	SPCP31B10.08c
313	SPBP23A10.11c	338	SPCC1682.09c	363	SPCC417.08	388	SPCPB16A4.03c
314	SPBP4G3.02	339	SPCC1682.14	364	SPCC4G3.02	389	SPNCRNA.01
315	SPBP4H10.13	340	SPCC16C4.12	365	SPCC548.06c	390	SPNCRNA.03
316	SPBP4H10.14c	341	SPCC16C4.13c	366	SPCC576.07	391	SPNCRNA.100
317	SPBP8B7.02	342	SPCC16C4.19	367	SPCC576.08c	392	SPNCRNA.1015
318	SPBP8B7.03c	343	SPCC1739.13	368	SPCC576.10c	393	SPNCRNA.1018
319	SPBP8B7.04	344	SPCC1753.03c	369	SPCC576.11	394	SPNCRNA.1032
320	SPBP8B7.05c	345	SPCC1795.04c	370	SPCC576.12c	395	SPNCRNA.1042
321	SPBP8B7.06	346	SPCC1795.05c	371	SPCC5E4.07	396	SPNCRNA.1055
322	SPBP8B7.07c	347	SPCC1795.06	372	SPCC61.01c	397	SPNCRNA.1070
323	SPBP8B7.15c	348	SPCC18.13	373	SPCC613.04c	398	SPNCRNA.1072
324	SPBP8B7.16c	349	SPCC18.14c	374	SPCC613.05c	399	SPNCRNA.1083
325	SPBP8B7.32	350	SPCC1827.03c	375	SPCC613.06	400	SPNCRNA.110

No.	Systematic ID	No.	Systematic ID	No.	Systematic ID	No.	Systematic ID
401	SPNCRNA.1103	426	SPNCRNA.1464	451	SPNCRNA.1695	476	SPNCRNA.472
402	SPNCRNA.113	427	SPNCRNA.1465	452	SPNCRNA.1696	477	SPNCRNA.477
403	SPNCRNA.1136	428	SPNCRNA.1471	453	SPNCRNA.1698	478	SPNCRNA.525
404	SPNCRNA.1140	429	SPNCRNA.1487	454	SPNCRNA.171	479	SPNCRNA.532
405	SPNCRNA.119	430	SPNCRNA.1490	455	SPNCRNA.1710	480	SPNCRNA.579
406	SPNCRNA.1197	431	SPNCRNA.1510	456	SPNCRNA.1711	481	SPNCRNA.625
407	SPNCRNA.1203	432	SPNCRNA.1511	457	SPNCRNA.1712	482	SPNCRNA.626
408	SPNCRNA.1245	433	SPNCRNA.1517	458	SPNCRNA.197	483	SPNCRNA.627
409	SPNCRNA.1278	434	SPNCRNA.1524	459	SPNCRNA.219	484	SPNCRNA.628
410	SPNCRNA.128	435	SPNCRNA.1545	460	SPNCRNA.242	485	SPNCRNA.639
411	SPNCRNA.1309	436	SPNCRNA.1573	461	SPNCRNA.254	486	SPNCRNA.648
412	SPNCRNA.132	437	SPNCRNA.1578	462	SPNCRNA.255	487	SPNCRNA.659
413	SPNCRNA.1358	438	SPNCRNA.1597	463	SPNCRNA.258	488	SPNCRNA.69
414	SPNCRNA.1359	439	SPNCRNA.1598	464	SPNCRNA.265	489	SPNCRNA.717
415	SPNCRNA.1375	440	SPNCRNA.1622	465	SPNCRNA.270	490	SPNCRNA.719
416	SPNCRNA.1389	441	SPNCRNA.1626	466	SPNCRNA.317	491	SPNCRNA.724
417	SPNCRNA.1390	442	SPNCRNA.1627	467	SPNCRNA.358	492	SPNCRNA.743
418	SPNCRNA.1397	443	SPNCRNA.163	468	SPNCRNA.399	493	SPNCRNA.756
419	SPNCRNA.143	444	SPNCRNA.1643	469	SPNCRNA.400	494	SPNCRNA.770
420	SPNCRNA.144	445	SPNCRNA.1651	470	SPNCRNA.403	495	SPNCRNA.781
421	SPNCRNA.1443	446	SPNCRNA.1665	471	SPNCRNA.419	496	SPNCRNA.783
422	SPNCRNA.1444	447	SPNCRNA.1671	472	SPNCRNA.425	497	SPNCRNA.784
423	SPNCRNA.1448	448	SPNCRNA.1672	473	SPNCRNA.445	498	SPNCRNA.806
424	SPNCRNA.1457	449	SPNCRNA.1673	474	SPNCRNA.467	499	SPNCRNA.807
425	SPNCRNA.1459	450	SPNCRNA.1674	475	SPNCRNA.471	500	SPNCRNA.812

No.	Systematic ID	No.	Systematic ID	No.	Systematic ID
501	SPNCRNA.82	526	SPNCRNA.965	551	SPSNORNA.42
502	SPNCRNA.826	527	SPNCRNA.980	552	SPSNORNA.44
503	SPNCRNA.831	528	SPNCRNA.989	553	SPSNORNA.45
504	SPNCRNA.861	529	SPNCRNA.990	554	SPSNRNA.04
505	SPNCRNA.862	530	SPRRNA.26		
506	SPNCRNA.863	531	SPRRNA.37		
507	SPNCRNA.87	532	SPRRNA.42		
508	SPNCRNA.870	533	SPSNORNA.03		
509	SPNCRNA.872	534	SPSNORNA.04		
510	SPNCRNA.873	535	SPSNORNA.05		
511	SPNCRNA.893	536	SPSNORNA.06		
512	SPNCRNA.9001	537	SPSNORNA.07		
513	SPNCRNA.902	538	SPSNORNA.08		
514	SPNCRNA.903	539	SPSNORNA.09		
515	SPNCRNA.918	540	SPSNORNA.10		
516	SPNCRNA.92	541	SPSNORNA.11		
517	SPNCRNA.93	542	SPSNORNA.18		
518	SPNCRNA.933	543	SPSNORNA.20		
519	SPNCRNA.942	544	SPSNORNA.21		
520	SPNCRNA.944	545	SPSNORNA.22		
521	SPNCRNA.949	546	SPSNORNA.26		
522	SPNCRNA.950	547	SPSNORNA.29		
523	SPNCRNA.954	548	SPSNORNA.32		
524	SPNCRNA.957	549	SPSNORNA.33		
525	SPNCRNA.963	550	SPSNORNA.41		

Supplementary Table S4. Gene ontology showing each cluster

GOID	Term	p-value	Num_list_Annotation
GO:0002181	cytoplasmic translation	7.16E-28	130
GO:0006412	translation	3.30E-16	132
GO:0043043	peptide biosynthetic process	1.22E-15	132
GO:0006518	peptide metabolic process	6.86E-15	133
GO:0043604	amide biosynthetic process	3.33E-14	135
GO:0042254	ribosome biogenesis	1.48E-13	86
GO:0043603	cellular amide metabolic process	1.33E-12	138
GO:0042255	ribosome assembly	2.41E-11	27
GO:0022613	ribonucleoprotein complex biogenesis	7.48E-10	90
GO:1901566	organonitrogen compound biosynthetic process	1.33E-09	171
GO:0000028	ribosomal small subunit assembly	2.55E-08	13
GO:0042273	ribosomal large subunit biogenesis	8.79E-08	27
GO:0000027	ribosomal large subunit assembly	3.68E-06	14
GO:XXXXXXX	unannotated	8.26E-122	145

Supplementary Table S5. Genes with both repressed transcription and reduced Atf1 binding

no.	Systematic ID	ribosome related gene
1	SPAPB15E9.02c	
2	SPBC14F5.05c	
3	SPBC27.08c	
4	SPAC19B12.02c	
5	SPAC926.04c	
6	SPBP8B7.16c	
7	SPBC12C2.07c	
8	SPAC664.06	✓
9	SPAC8E11.10	
10	SPAC19G12.05	
11	SPAC3H5.05c	✓
12	SPBC19G7.04	
13	SPBP4G3.02	
14	SPAC1250.05	✓
15	SPAC57A10.12c	
16	SPAC1071.10c	
17	SPBC8E4.01c	
18	SPCC18.13	
19	SPBC16C6.12c	✓
20	SPCC1183.07	✓
21	SPAC140.02	✓
22	SPAC11E3.13c	
23	SPAC110.01	
24	SPAC890.05	✓
25	SPAC10F6.01c	
26	SPBC1105.05	
27	SPBC359.03c	
28	SPCC1739.13	
29	SPBC26H8.06	
30	SPAC1F8.07c	
31	SPAC1039.02	
32	SPAC110.04c	
33	SPAC12G12.04	
34	SPBC30D10.18c	✓

1. Takemata,N., Oda,A., Yamada,T., Galipon,J., Miyoshi,T., Suzuki,Y., Sugano,S., Hoffman,C.S., Hirota,K. and Ohta,K. (2016) Local potentiation of stress-responsive genes by upstream noncoding transcription. *Nucleic Acids Res.*, **44**, 5174–5189.

2. Asada,R., Takemata,N., Hoffman,C.S., Ohta,K. and Hirota,K. (2015) Antagonistic controls of chromatin and mRNA start site selection by Tup family corepressors and the CCAAT-binding factor. *Mol. Cell. Biol*, **35**, 847–855.

3. Tashiro,S., Asano,T., Kanoh,J. and Ishikawa,F. (2013) Transcription-induced chromatin association of RNA surveillance factors mediates facultative heterochromatin formation in fission yeast. *Genes Cells*, **18**, 327–339.

4. Hiriart,E., Vavasseur,A., Touat-Todeschini,L., Yamashita,A., Gilquin,B., Lambert,E., Perot,J., Shichino,Y., Nazaret,N., Boyault,C., *et al.* (2012) Mmi1 RNA surveillance machinery directs RNAi complex RITS to specific meiotic genes in fission yeast. *EMBO J.*, **31**, 2296–2308.
